# Associations of parental age with health and social factors in adult offspring. Methodological pitfalls and possibilities

**DOI:** 10.1038/srep45278

**Published:** 2017-03-27

**Authors:** David Carslake, Per Tynelius, Gerard van den Berg, George Davey Smith, Finn Rasmussen

**Affiliations:** 1 MRC Integrative Epidemiology Unit at the University of Bristol, Bristol, UK; 2 School of Social and Community Medicine, University of Bristol, Bristol, UK; 3 Department of Public Health Sciences, Karolinska Institute, Stockholm, Sweden; 4 School of Economics, Finance and Management, University of Bristol, Bristol, UK; 5 Department of Health Sciences, Lund University, Sweden

## Abstract

Parental age is increasing rapidly in many countries. Analysis of this potentially important influence on offspring well-being is hampered by strong secular trends and socioeconomic patterning and by a shortage of follow-up data for adult offspring. We used Swedish national data on up to 3,653,938 offspring to consider the associations of parental age with a suite of outcomes in adult offspring, comparing the results from an array of statistical methods for optimal causal inference. The offspring of older mothers had higher BMI, blood pressure, height, intelligence, non-cognitive ability and socioeconomic position. They were less likely to smoke or to be left-handed. Associations with paternal age were strongly, but not completely, attenuated by adjustment for maternal age. Estimates from the commonly-used sibling comparison method were driven primarily by a pathway mediated by offspring date of birth when outcomes showed strong secular trends. These results suggest that the intra-uterine and early life environments provided by older mothers may be detrimental to offspring cardiovascular health, but that their greater life experience and social position may bring intellectual and social advantages to their offspring. The analysis of parental age presents particular challenges, and further methodological developments are needed.

The average age of parents at the birth of their offspring is increasing in most developed countries^1^. This demographic shift has potential consequences not only for the perinatal and childhood health of the offspring^2^,^3^,^4^ but also on their adult health. Various mechanisms may be responsible for a causal effect of parental age on offspring outcomes.

Rates of genetic mutation in germ cells are expected to increase with age in fathers, but not mothers^5^. This is because oocytes undergo only about 23 divisions per generation, of which only one occurs after the mother’s puberty, whereas spermatogonia undergo 30 divisions before puberty and continue to divide approximately 23 times per year afterwards^6^,^7^. The rate of meiotic chromosome segregation problems increases with age in men and women^8^,^9^. The association with age, and the proportion of the resulting offspring aneuploidy that is attributed to each parent, varies from one chromosome to another but the majority (88% of trisomies in one study^8^) are maternal in origin. In contrast to genetic mutation, the intra-uterine consequences of advanced parental age are likely to be restricted to maternal age. Such intra-uterine processes may nonetheless result in an observed association between paternal age and offspring outcomes because of the strong correlation between maternal and paternal age. The disentanglement of all maternal and paternal age effects on offspring outcomes is an important methodological challenge. Economic circumstances generally improve with age, so older parents might provide a better childhood environment^10^. Conversely, they are also likely to die earlier in their offspring’s life, which may deprive the offspring of social and financial support^11^. Birth order is another factor closely associated with parental age and which might influence offspring outcomes. Age-related changes in individual parents’ economic circumstances might mediate causal effects of parental age on offspring outcomes, but variation among parents in their lifelong average SEP probably confounds the association between them, influencing both family planning decisions and the health of the offspring to generate an association between them that does not represent a causal effect of parental age on offspring outcomes.

Associations of parental age with offspring adult health have been less studied than those with perinatal and childhood health, perhaps because of the length of follow-up required. In this study, we use Swedish national data to examine associations between parental age and adult offspring outcomes, with offspring birth weight and length included as additional outcomes to aid in the interpretation of the adult outcomes. Parental age data are available from a sufficiently early date to allow the analysis of outcomes in adult offspring, and the size of the data set gives sufficient power to compare the associations with maternal and paternal age. Previous studies of the adult consequences of parental age have studied single outcomes, or small groups of outcomes with a close thematic link. We instead study the full array of outcomes for which data were available, and which are plausibly related to adult cardiovascular health or social and intellectual success. Studying many outcomes with comparable methodology in the same data allows us to look for consistent patterns in offspring characteristics as a function of parental age among related outcomes.

## Results

Table 1 shows sample sizes from the primary and sibling-comparison analyses and average values among sons and their parents used in the primary analysis, for each outcome or other variable associated with parental age. Mean paternal age was 3.1 years greater than mean maternal age and the correlation between them was 76.1%. The number of paternal families was slightly lower than the number of maternal families. The equivalent information for daughters is shown in the supplement (Supplementary Table S1). Estimates of mean parental age according to factors which might affect it (Supplementary Fig. S1) showed that the rapid and consistent increase in average parental age since about 1970 is reversing an equally rapid and consistent decline which took place before 1970. They also show a U-shaped association between mean parental age and parental occupational and educational SEP, with parents of intermediate SEP being youngest on average. Parents with missing or “other” occupational SEP were particularly old at their son’s birth, while those with missing education data, particularly mothers, were younger parents. Most outcomes showed approximately linear trends over the course of the study (Supplementary Fig. S2), but DBP had a U-shaped trend, mirroring the trend in parental age. Non-cognitive ability increased slowly from 1951 to about 1964, after which values were considerably elevated. Rates of non-manual employment fell considerably over the study period, perhaps due to more recent subjects still being at a relatively early career stage when employment was last recorded in 1990.

The primary analyses with default adjustment (set (e): offspring DOB and birth order; maternal and paternal occupational and educational SEP; and other parent’s age at offspring birth) showed that the offspring of older mothers were born lighter and shorter. As adults they were taller, had higher SBP, DBP, intelligence and non-cognitive ability, were of higher educational and occupational SEP and were less likely to smoke or to be left-handed (Table 2). Most of the equivalent associations with paternal age (Table 3) were in the same direction, but considerably weaker than those for maternal age; a difference due in large part to a substantial attenuation of the paternal age associations when they were adjusted for maternal age (but not vice versa; compare adjustment sets (d) and (e) in Supplementary Tables S4 and S5). Adjustment set (f), in which the linear adjustment for the other parent’s age was replaced by categories, gave very similar results to set (e) and is not shown. Adjustment for birth order (compare adjustment sets (d) and (c) in Supplementary Table S4) led to a considerable amplification of the associations with all outcomes except for birth weight and length, which changed from positive associations to negative ones when adjusted for birth order. Offspring BMI was weakly negatively associated with maternal age before adjustment for SEP and birth order, and weakly positively associated afterwards. Associations with paternal age were similar but weaker (Supplementary Table S5).

**Figure 1:**
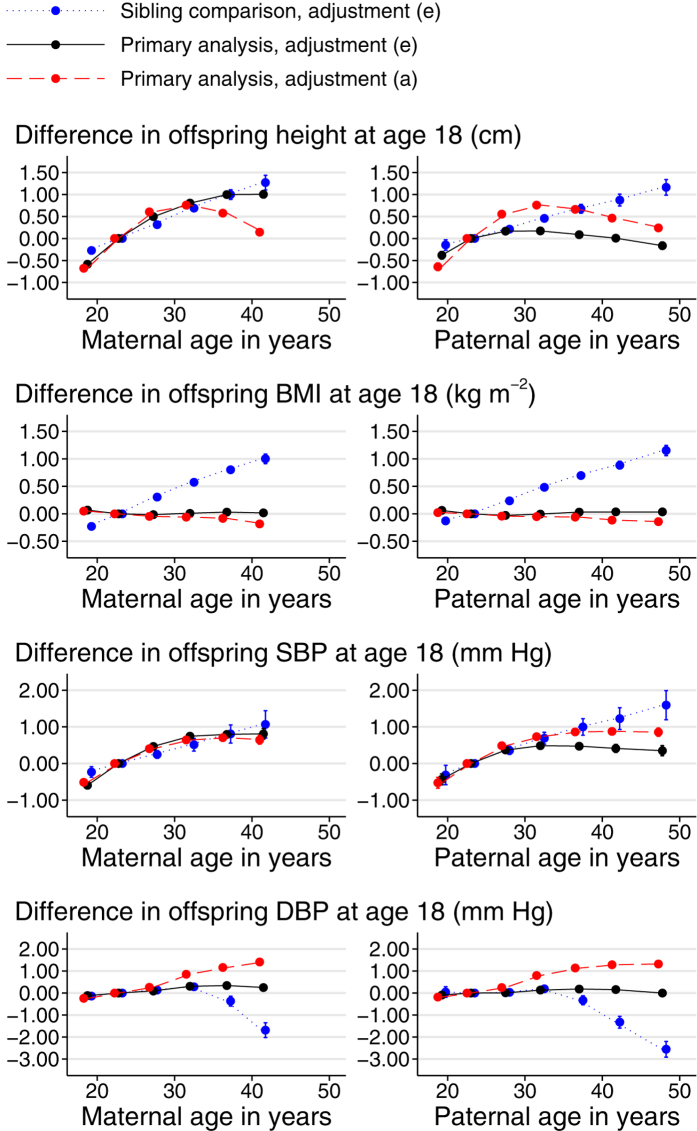
Associations between outcomes in sons and parental age at son’s birth. Parental age in years was put into classes of <20, 20–24 (reference), 25–29, 30–34, 35–39, 40–44 and ≥45 and each class was plotted at its median. Points, but not connecting lines, are transposed horizontally by +/−0.5 years for clarity. The two oldest classes were combined for maternal age. Error bars are 95% confidence intervals. Primary analysis associations are shown with adjustment (**a**) (red, dashed line; no adjustment) and (**e**) (black, solid line; adjustment for offspring DOB, parental SEP, offspring birth order and the other parent’s parental age). Sibling comparison analyses (blue, dotted line) are shown with adjustment (**e**) (offspring birth order and the other parent’s SEP and parental age). Offspring of both sexes were used in the analysis of offspring sex.

**Figure 2:**
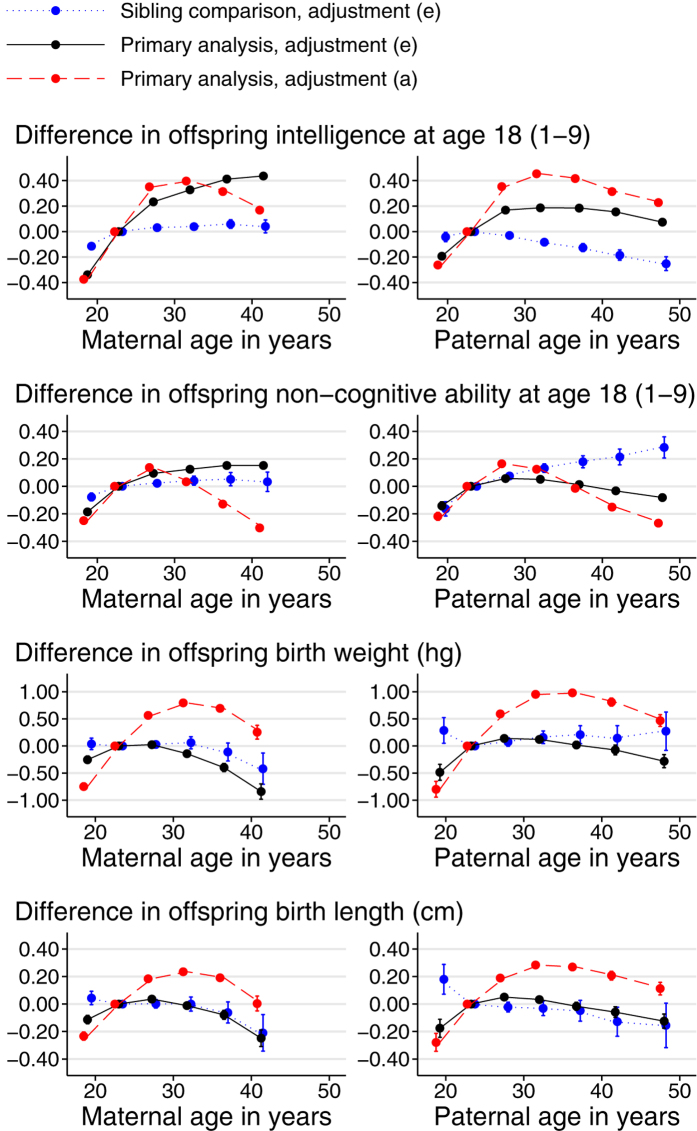
Associations between outcomes in sons and parental age at son’s birth (continued). Parental age in years was put into classes of <20, 20–24 (reference), 25–29, 30–34, 35–39, 40–44 and ≥45 and each class was plotted at its median. Points, but not connecting lines, are transposed horizontally by +/−0.5 years for clarity. The two oldest classes were combined for maternal age. Error bars are 95% confidence intervals. Primary analysis associations are shown with adjustment (**a**) (red, dashed line; no adjustment) and (**e**) (black, solid line; adjustment for offspring DOB, parental SEP, offspring birth order and the other parent’s parental age). Sibling comparison analyses (blue, dotted line) are shown with adjustment (**e**) (offspring birth order and the other parent’s SEP and parental age). Offspring of both sexes were used in the analysis of offspring sex.

**Figure 3:**
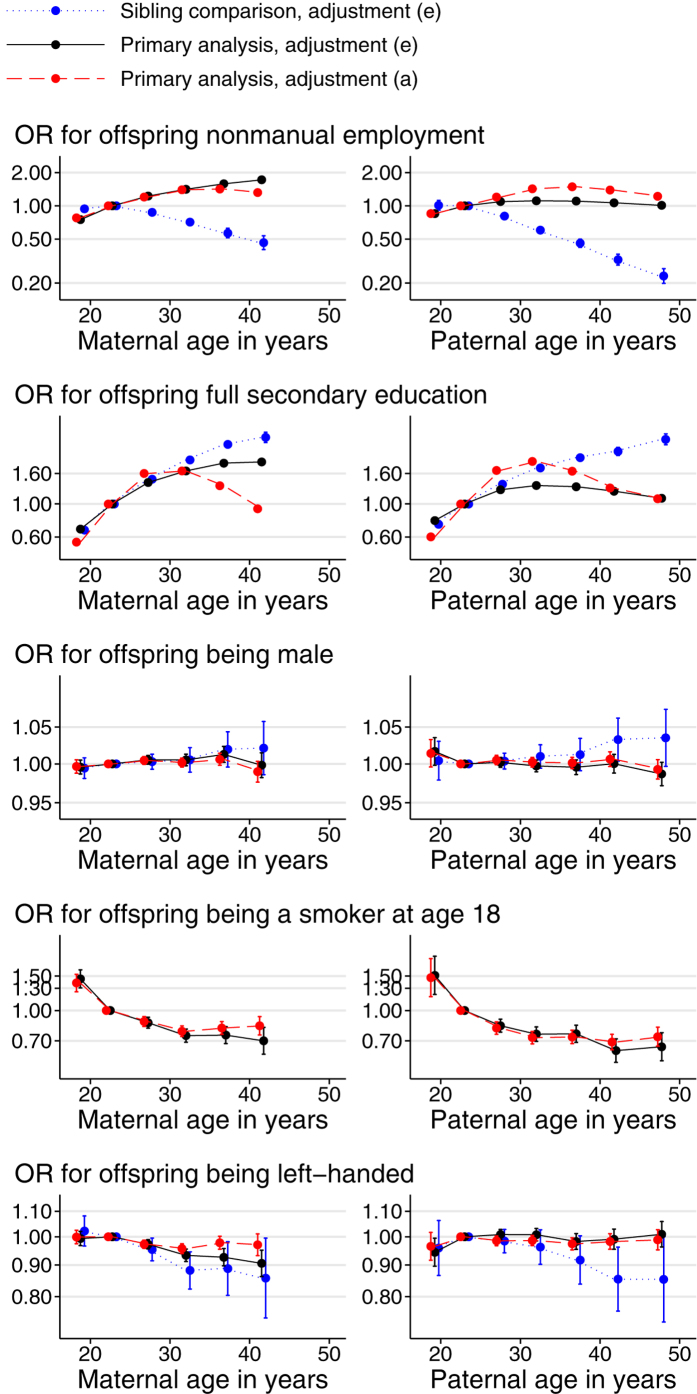
Associations between outcomes in sons and parental age at son’s birth (continued). Parental age in years was put into classes of <20, 20–24 (reference), 25–29, 30–34, 35–39, 40–44 and ≥45 and each class was plotted at its median. Points, but not connecting lines, are transposed horizontally by +/−0.5 years for clarity. The two oldest classes were combined for maternal age. Error bars are 95% confidence intervals. Primary analysis associations are shown with adjustment (**a**) (red, dashed line; no adjustment) and (**e**) (black, solid line; adjustment for offspring DOB, parental SEP, offspring birth order and the other parent’s parental age). Sibling comparison analyses (blue, dotted line) are shown with adjustment (**e**) (offspring birth order and the other parent’s SEP and parental age). Offspring of both sexes were used in the analysis of offspring sex.

Examination of the plots by categories of parental age (Figs 1, 2, 3) shows that increases in offspring height, intelligence, non-cognitive ability and SEP with parental age levelled off (when adjusted) or reversed (unadjusted) after a parental age of around 30. The positive association of offspring SBP with parental age levelled off for the oldest parents regardless of adjustment while associations of offspring DBP with parental age were closer to linearity. When adjusted, birth weight and length increased sharply with parental age, peaking for parents in their late twenties. They then declined sharply with maternal age and more gradually with paternal age. Some outcomes, most notably BMI and non-manual employment, showed sibling-comparison results which were strikingly different in shape and magnitude from the equivalent primary analysis results.

Sample sizes were considerably reduced in the sibling-comparison analyses (Table 1), particularly for binary outcomes. Linear results from the primary and sibling-comparison analyses of maternal age were mostly in the same direction, but were often of very different magnitude (Table 2). Those for paternal age differed considerably in both direction and magnitude (Table 3). When the primary analyses were adjusted for parental DOB in place of offspring DOB, however, there was a much closer match to the sibling-comparison analyses. This match was not greatly improved by restricting the data to those used in the sibling-comparison analyses (Tables 2 and 3).

The primary associations of parental age with most outcomes did not differ substantially between sons who were the oldest sibling, and sons who were not (Supplementary Tables S6 and S7), although the confidence intervals were sufficiently narrow to demonstrate that the minor differences between the groups were not due to chance in most cases. In the two-variable analysis, associations for maternal age at the index offspring’s birth were generally close to the associations found in the primary analysis restricted to later offspring (Supplementary Table S6). The negative associations with birth length and weight, and the positive association with adult BMI, were considerably amplified. The equivalent associations with paternal age were often greatly amplified and sometimes opposite in direction from those in the primary analysis of later offspring (Supplementary Table S7). Associations with maternal or paternal age at their first offspring’s birth in the two-variable model differed substantially from those for parental age at the index offspring’s birth, both in the two-variable analysis and in the restricted primary analysis.

The linear primary analyses showed no evidence that offspring sex was associated with parental age (Supplementary Table S2), though there was a weak suggestion of a female bias in the offspring of the oldest mothers (Fig. 3). The odds of being in non-manual employment increased with increasing maternal or paternal age slightly more for daughters (Supplementary Tables S2 and S3) than for sons (Tables 2 and 3). The magnitude of these differences was small, however, and for the other outcomes available in daughters the confidence intervals overlapped with those from the estimates made among sons.

Most associations with maternal age (fully adjusted including for offspring DOB) were in the same direction among sons born before and after the end of 1969, with some alterations in magnitude (Supplementary Table S8). Maternal age was positively associated with BMI in the earlier period (when parental age was falling) and negatively associated in the second period (when parental age was rising). Associations with paternal age differed greatly between the two periods (Supplementary Table S9). Similar analyses using the sibling-comparison method (Supplementary Tables S10 and S11) had relatively wide confidence intervals, but some associations with parental age changed considerably between the two periods, and those for DBP and non-cognitive ability clearly changed direction.

In the subset of data for which parental survival to the offspring’s 16^th^ birthday could be calculated (Supplementary Tables S12 and S13), associations between offspring outcomes and parental age were very similar to those in the full data (Tables 2 and 3). Additional adjustment for lifespan overlap made very little difference to the associations.

Definition of sibling groups by both parents’ identities instead of by the identity of the “exposure” parent had a negligible effect on the primary analyses (Supplementary Tables S18 and S19). In the sibling comparison analyses of maternal age, most associations were a little reduced (i.e. became less positive, or more negative) but those for DBP, intelligence and non-cognitive ability were substantially reduced. Changes to the sibling comparison analyses of paternal age were similar in magnitude for each outcome, but inconsistent in direction.

Adjustment for offspring birth weight and birth length had very little effect on the primary or sibling comparison analyses (Supplementary Tables S14–S17), but restriction of the study sample to those with data available on these variables changed estimates substantially, particularly in the sibling comparison analysis for DBP and non-cognitive ability (outcomes with strong nonlinear secular trends).

## Discussion

### Interpretation of the different analyses

**Figure 4:**
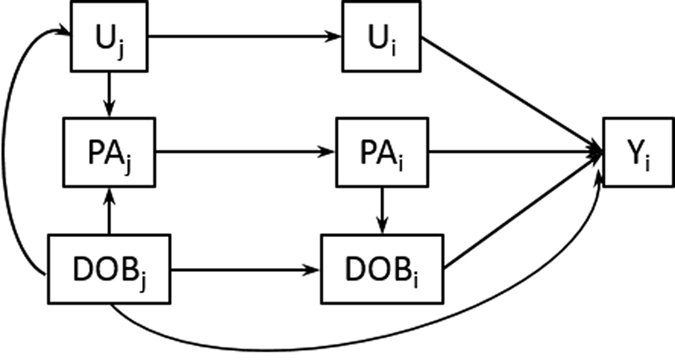
Directed Acyclic Graph. Offspring outcomes (Y_i_) may be associated with parental age at the birth of their offspring (PA_i_), the date of birth of the parent (DOB_j_) and offspring (DOB_i_), and unmeasured confounders at the parent (U_j_) and offspring (U_i_) level. PA_j_ is the parent-level inclination towards earlier or later parenthood. Note that any one of PA_i_, DOB_i_ and DOB_j_ is uniquely identified by the other two.

When inferring whether the ongoing rise in mean parental age will lead to changes in outcomes for the offspring, it is necessary to determine, as far as possible, whether the observed associations are due to a causal effect of parental age on offspring outcomes, or if both are influenced by a confounding factor. In the within-family context, the causal effect of parental age on offspring outcomes includes an indirect effect mediated by the offspring’s DOB^12^ (Fig. 4). This depends on the secular trend in outcome at the time, and is thus of lesser interest and should ideally be distinguished from direct effects of parental age. Having only observational data, we conducted numerous sensitivity analyses to try and distinguish causal from confounded associations, and direct ones from those mediated by offspring DOB.

The obvious first approach to remove confounding and mediation is to adjust for the relevant measured variables. For example, the attenuation of most associations with paternal age when adjusted for maternal age (adjustment sets (e) vs (d) in Supplementary Table S5) suggests that they are largely due to confounding or mediation by maternal age. In the primary analyses, adjustment for offspring DOB allows us to assess the importance of the pathway from parental age to offspring outcomes mediated by secular trends in the outcome (adjustment sets (a) vs (b) in Supplementary Tables S4 and S5), suggesting that it is particularly important for BMI, DBP and occupational SEP; outcomes with very strong secular trends (Supplementary Fig. S2).

The big disadvantage with the adjusted primary analysis is that it can only account for measured variables. A sibling comparison analysis has the attraction of adjusting for all family-level confounders, whether or not they have been measured^13^. However, this approach also has a number of drawbacks which need to be considered when interpreting its results. First, the analysis is conducted on the subpopulation of offspring who have (same sex) siblings. For binary outcomes, those siblings must also have discordant outcomes. Repetition of the primary analyses on the restricted data set (Tables 2 and 3) allows us to conclude that this is a minor influence for most outcomes, but influential for birth weight, educational SEP and left-handedness in relation to maternal age. When sibling groups were defined by both parents’ identities (Supplementary Tables S18 and S19), those offspring who only had half-siblings were also excluded from the sibling comparison analysis (e.g. for BMI and maternal age, N decreased from 764,329 to 695,536). The exclusion of this potentially atypical subset of the data was probably responsible for changes in the sibling comparison analyses, which applied particularly to those outcomes with strong nonlinear secular trends. Second, a sibling comparison analysis can increase, rather than decrease, bias from an individual-level confounder if it is less strongly correlated among siblings than the exposure is^14^,^15^. Even though parental age is necessarily different among non-twin siblings, it was nonetheless strongly correlated among siblings (maternal identity accounting for 38.4% of the variance in maternal age in an unadjusted variance components analysis). However, individual-level confounders of parental age and offspring outcomes are likely to be rare, because parental age is determined at the offspring’s birth (and approximately determined at their conception), and is thus unlikely to be influenced by offspring-specific variables. Birth order might be considered an individual-level confounder of parental age and outcomes, but was measured and can be adjusted for.

Third, the sibling comparison procedure adjusts for confounding by secular trends of family-level parental age and offspring outcomes, but it cannot adjust for mediation by secular trends of the effect of individual-level parental age on offspring outcomes because offspring DOB and parental age are perfectly correlated within families. The commonly used practice of adjusting for year of birth (which is merely DOB with reduced precision) in a sibling-comparison analysis of parental age^12^,^13^,^16^ does not solve this issue and may lead to additional confounding (Supplementary Note online). If the pathway mediated by DOB were the only association between parental age and the outcomes (i.e. no direct effect and no individual-level confounding), we would expect the sibling-comparison association to equal the individual-level secular trend in the outcome and the primary association to be null. It is therefore likely that the mediated pathway is highly influential in the associations of BMI and DBP with parental age (Tables 2 and 3). DBP and non-cognitive ability both have secular trends that change in trajectory in about 1970 (Supplementary Fig. S2), and the very different sibling-comparison results obtained for these two outcomes before and after this date (Supplementary Tables S10 and S11) support a strong role for mediation by DOB in these cases. The sibling comparison analysis and the primary analysis adjusted for parental DOB in place of offspring’s DOB (Tables 2 and 3) share the same mediated and direct effects of parental age on outcomes and differ primarily in that the latter retains family-level confounded pathways other than family-level confounding by secular trends. The similarity in the results from these two analyses suggests that family-level confounding other than by secular trends was not an important influence on these results for most outcomes. Possible exceptions are non-cognitive ability, birth weight, educational SEP and (for maternal age) left-handedness.

Fourth, attenuation due to measurement error in the exposure is greater for sibling-comparison than for conventional estimates^14^. Although DOB (and thus parental age) was probably measured with minimal error, its subsequent rounding may have biased estimates, particularly those from the sibling comparison method, towards the null. Finally, the selection of discordant exposures can bias a sibling comparison analysis^14^, but this does not apply here because parental age always differs between non-twin siblings.

When analyses are mutually adjusted for parental age at the birth of the first and index children, among later children, we can interpret the association with parental age at the birth of the first child as an effect of selection into delayed parenthood (i.e. family-level association, most of which is likely to be non-causal) and the association with parental age at the birth of the index child as an estimate which is adjusted for such family-level confounding^17^, although the adjustment does potentially open induced confounding pathways (Supplementary Note online). Adjustment for maternal age at the birth of the first child did not substantially change most associations with maternal age at the birth of the index child (Supplementary Table S6), suggesting once again that family-level confounding is limited. Nonetheless, there were clearly non-null independent associations with parental age at the birth of the first child, indicating associations between delayed family initiation and family-average offspring outcomes. For paternal age (Supplementary Table S7), adjustment for paternal age at the birth of the first child made substantial changes to a number of the associations, a result which may be linked to the strong co-linearity of paternal and maternal age and the attenuation of the primary associations with paternal age when adjusted for maternal age, but not vice versa.

### Comparison with the literature on early-life outcomes

Associations between parental age and early-life outcomes have been studied in rather more depth elsewhere, and we consider a limited selection of early-life outcomes here, primarily to aid the interpretation of adult outcomes. Most previous studies have found birth weight to be positively associated with maternal age^18^,^19^,^20^, often then declining again among the offspring of the oldest mothers^21^,^22^. These associations are generally attenuated by adjustment for socioeconomic position, and confounding by such factors has been suggested as an explanation for the associations^19^. We also found unadjusted associations between birth weight and maternal age that were inverse-U shaped with a particular reduction in the offspring of very young mothers. However, adjustment, particularly for birth order (Supplementary Table S4), greatly attenuated the disadvantage of low maternal age but not advanced maternal age (Fig. 2). The sibling-comparison analysis found a weaker negative association than the similarly adjusted primary analysis (Table 2), perhaps due to the steady increase in mean birth weight over the period of the study.

There is some evidence in the literature that left-handedness is more common in the offspring of older mothers^23^,^24^, perhaps due to their increased risk of prenatal and perinatal stress^25^, although these claims remain controversial^24^,^26^. In contrast, in the primary analyses of the present study left handedness was weakly negatively associated with maternal age and had no association with paternal age (Tables 2 and 3). The sibling-comparison analyses found stronger negative associations. These were unlikely to be mediated by offspring DOB because left-handedness showed only a very slight increase over the period of the study. The weakly positive association with parental age at the oldest offspring’s birth (Supplementary Tables S6 and S7) suggests that the relative attenuation of the primary association compared to the sibling-comparison analysis may have been due to some unmeasured family-level confounding. Left-handedness may be associated with increased incidence of some autoimmune diseases^27^, educational and employment disadvantages^28^ and reduced longevity^29^, in which case reduced left-handedness in the offspring of older mothers could be viewed as advantageous in the present study, in contrast to the associations with birth weight and length.

Although there was no linear association between parental age and offspring sex, there was some evidence that the very oldest mothers (over 40) were more likely to have daughters. Pregnancy complications are more common in older mothers^30^, and are more likely to be fatal for male than for female foetuses^31^,^32^. This could account for the results observed here and in other several other studies^33^,^34^. Some authors have suggested that paternal age influences the proportions of X and Y-bearing sperm, and that maternal age is associated with offspring sex only by association with paternal age^35^,^36^. In our study though, the oldest fathers (over 45) showed a weaker association with a female-biased sex ratio after mutual adjustment than the oldest mothers did (Fig. 3). It has also been suggested that associations with parental age are driven by their association with maternal parity^37^, but the associations in the present study were not substantially attenuated by adjustment for parity. It should be noted that the study was restricted to offspring who were alive on 1^st^ July 1991, when they were between 3 and 59 years of age. The sex ratios presented here are therefore not sex ratios at birth, although the overall ratio of 1.053 males per female lies within the typical range of reported values for sex ratio at birth^33^,^34^, suggesting that male-biased mortality was minimal.

### Comparison with the literature on adult cardiovascular health

With adjustment set (e), of which DOB was the most influential, offspring BMI had a slightly U-shaped association with parental age, with the lowest BMI in the offspring of mothers in their 20 s (Fig. 1). The two-variable and sibling comparison analyses both suggested that the null overall linear associations of parental age with offspring BMI were due to a negative between-family association, probably due to family-level confounding, counteracted by a positive within-family association (amplified in the case of the sibling comparison analysis by a positive secular trend in BMI). A negative between-family association due to socioeconomic confounding might be particularly pronounced when the very youngest mothers are compared with the others, accounting for the increased average adult BMI of the offspring of mothers under 20 years old. The few previous studies to have examined the association between parental age and offspring BMI found null or conflicting results^38^. Studies of adult BMI are even rarer; the risk of obesity at 18 was positively associated with paternal, but not maternal age in a study of male Norwegian conscripts which adjusted for secular trends and SEP^38^.

The present results suggest clearly that SBP is positively associated with within-family maternal and paternal age. The primary and two-variable analyses suggest the same of DBP, but the sibling comparison analysis suggests a strongly negative within-family association, a result which is largely attributable to the strong secular trend in DBP. A meta-analysis of three studies of blood pressure at 5–7 years old^39^ found a positive association with maternal age overall (paternal age was neither tested nor adjusted for), but the association was weak and inconsistent between studies. A subsequent single large study of blood pressure at 15 years old^40^ found no association with maternal or paternal age. Neither study examined the shape of the associations.

Greater height is associated with improved cardiovascular health^41^. The present results supported a positive association between maternal age and offspring adult height, but the primary, two-variable and sibling comparison analyses disagreed regarding paternal age. The positive association was partially reversed among mothers in their late 30 s or early 40 s in the unadjusted primary analysis, but continued in the adjusted primary or sibling comparison analyses. A previous analysis^12^ of maternal age using a subset (born 1965–1977) of the present data found similar results except for their sibling-comparison analysis adjusted for year of birth, which we consider unreliable (Supplementary Note online). A small (N = 277) study^42^,^43^ of New Zealand children aged 3–10 and of relatively uniform socioeconomic background found that offspring height was independently positively associated with maternal and paternal age after adjustment for age, sex, birth weight, birth order and mid-parental height; a result the authors considered likely to persist into adulthood.

### Comparison with the literature on adult social & intellectual status

In unadjusted analyses, we found inverse-U shaped associations of offspring intelligence, non-cognitive ability and educational attainment with parental age, such that the offspring of the youngest and oldest parents were disadvantaged (Figs 2 and 3). This is consistent with most previous studies of offspring intelligence^44^,^45^,^46^, educational achievement^12^,^13^,^47^ and social adjustment^48^. There is less consistency across studies once adjustment is attempted, due perhaps in part to the different approaches taken.

With adjustment set (e), we found that intelligence remained lower in the offspring of young mothers, but was no longer reduced in the offspring of older mothers (Fig. 2). The sibling-comparison analysis gave a similar result. This is consistent with two previous studies^45^,^46^, although two others^44^,^49^ found least some decline in offspring intelligence among older mothers as well as younger ones. Most of these studies found that associations of maternal and paternal age with offspring intelligence were similar, both before and after adjustment. We found that adjustment in the primary analyses did not entirely attenuate the reduced intelligence among offspring of older fathers, and this result was even more pronounced in the sibling-comparison study, which can only partly be attributed to the slight negative trend in intelligence over the study. This might suggest that the sibling-comparison analysis is avoiding some unmeasured confounding which was masking the detrimental effects of advanced paternal age in the primary analyses. Finally, an earlier subset (born 1951–1976) of the data used in the present study was used previously in a comprehensively adjusted sibling-comparison study^16^. This found no association with paternal age and reduced intelligence in the offspring of older mothers, but the inclusion of offspring year of birth among the confounders makes it difficult to compare these results confidently with the current ones (Supplementary Note online).

In the present analyses of offspring educational achievement, the effect of adjustment was similar to that in analyses of intelligence; educational achievement remained relatively low in the offspring of young parents, but the apparent disadvantage of having older parents was almost entirely attenuated. Similar mechanisms may be at work, and the adjustment for birth order may be particularly important here, since intelligence is negatively associated with birth order^50^,^51^, which in turn is positively associated with parental age. A previous study of the same data source, using a slightly different outcome and a more recent cohort (born 1973–1991)^13^ found similar results, except that adjustment also attenuated the apparent disadvantage of having a young father somewhat. Another Swedish study^52^ found reduced educational outcomes in the offspring of young fathers (attenuated by adjustment), but no decline (whether adjusted or not) among the offspring of older fathers. A recent review^53^ concluded that educational achievement was positively associated with maternal age, but that studies differed in the extent to which that association was attenuated by adjustment for socioeconomic position. A study conducted in five low and middle income countries^47^ found that after adjustment for factors including socioeconomic status, educational completion rates rose almost linearly with maternal age (prior to adjustment they had been highest at intermediate maternal age).

The present analysis of non-cognitive ability may be compared with a study of social functioning in Israeli men aged 16–17^48^. Both studies found inverted U-shaped associations with paternal age in unadjusted data which remained, albeit attenuated, after similar levels of adjustment (Fig. 2). Adjustment of the inverted U-shape associations with maternal age, however, emphasised the disadvantages of high maternal age in the earlier study, but low maternal age in the present study. Sibling-comparison associations in the present study were closer to linearity and to the null for maternal age, but reversed the apparent disadvantages of advanced paternal age found in primary analyses.

A small and unadjusted study of Swedish men^54^ found that maternal age was greater among those that did not smoke in late adolescence, which agrees with our findings (Table 2).

### Why does parental age associate with offspring adult outcomes?

Although the magnitude varied, there was consistent evidence across the primary, sibling comparison and two-variable analyses that the sons of older mothers are born smaller and lighter, but as adults are taller, more educated and intelligent, have higher non-cognitive ability and SBP and are less likely to be left-handed or to smoke. The sibling-comparison analysis and the two-variable analysis both suggested a modest positive association between parental age and offspring adult BMI (the sibling comparison result being greatly amplified by the positive secular trend), which is attenuated almost to the null in the primary analysis by a negative between-family association. Results from the primary and two-variable analyses suggested that the sons of older mothers also have higher occupational SEP and DBP, but results from the sibling-comparison analysis were in the opposite direction (probably due to the secular trends in these variables).

While some associations with greater parental age were advantageous and others disadvantageous to offspring, they were largely (left-handedness being the possible exception) consistent with a poorer intra-uterine environment, but a more resource-rich early-life environment. A child’s mother is uniquely responsible for its intrauterine environment, and increasing maternal age is associated with an increased risk of prenatal or perinatal complications^55^,^56^. The association with birth weight was more pronounced for maternal than paternal age, particularly after mutual adjustment, as we would expect from an intra-uterine effect. However, the weak negative association with birth length was similar for maternal and paternal age, before and after mutual adjustment, suggesting that some other mechanism may be responsible for this variable’s association with parental age. An adverse intrauterine environment in older mothers, as well as reducing birth weight, may lead to a “thrifty” phenotype in the offspring, which is associated with the components of the metabolic syndrome^57^,^58^. Maternal age has been shown to leave an epigenetic signature on offspring, which persists into adulthood^59^. This “thrifty” phenotype alone might explain the increased risk of adult adiposity and hypertension in the offspring of older mothers although it should be noted that if these are effects of the intrauterine environment, those effects do not appear to be mediated by the offspring’s birth weight or birth length (Supplementary Table S16). Additionally, however, people tend to gain in wealth and experience as they age, meaning that older parents potentially provide a more resource-rich environment for their offspring^53^,^60^. Such an environment could counteract the lower birth weight and length of children born to older parents to produce heavier and more hypertensive adults. Conversely in terms of health, it could also improve offspring intellectual development, leading to higher adult SEP and a reduced tendency to smoke. A notable feature of our results is that associations with paternal age were largely attenuated by adjustment for maternal age, suggesting that maternal age is the more important factor, and that paternal age covaries with offspring outcomes largely by association with maternal age. A family’s financial circumstances would be expected to depend at least as much, if not more, on the father’s age than the mother’s if fathers tended to be the main breadwinners of the family. Conversely, if it is the life experience of older parents that provides the more resource-rich environment for their offspring, then the association with maternal age would be stronger than the association with paternal age if mothers were disproportionately responsible for the childcare in the family. It may then be that the increased experience of older parents is more important to their offspring’s health than their increased wealth. The advantages and disadvantages of increasing parental age observed here remained relatively constant for parental ages exceeding the mid-30s (Fig. 1). A maternal age-induced “thrifty” phenotype or the increased life experience of older mothers are unlikely to reach a threshold at this age. It could be that beyond this age, the positive effects of parental age begin to be cancelled out by harmful biological processes in the male and female germ lines such as chromosome mis-segregation or increased mutation rates. If this were the case, however, we might expect the negative effects of parental age (on BMI, SBP and DBP) to accelerate after this age when they in fact also level off. It may simply be that an adult’s character and socioeconomic position (and thus the environment they provide for their children) are more stable after they reach their mid-30s, reducing the rate at which further increases in parental age affect offspring outcomes. The two outcomes in which the negative associations of parental age do accelerate after the mid-30s are the two which cannot be affected by the post-natal environment provided by the parents; birth weight and birth length.

If a pre-natal biological process such as germ-line mutation were influencing associations between parental age and offspring outcomes, then false paternity could further decrease the contribution of paternal, relative to maternal, age. However, this would not apply to post-natal mechanisms where the social father is more relevant than the genetic father. Furthermore, the modern rate of false paternity in developed countries, although rather uncertain, is probably substantially less than 10%^61^; not enough to account alone for the greater influence of maternal age on offspring outcomes after mutual adjustment.

The associations between offspring outcomes and paternal age were greatly reduced in magnitude after adjustment for maternal age, but were not attenuated to the null. We would not expect a maternal age effect on the intra-uterine environment to apply at all to paternal age, but an effect of parental life experience on the childhood environment might apply independently, but in weakened form, to paternal age. Alternatively, there may be some different, exclusively male, mechanism at work in addition to the effects of maternal age. Telomere length decreases with age in most proliferating cell types, but increases in sperm. It has therefore been suggested that older fathers (but not mothers) pass on longer telomeres to their offspring. This would predispose the offspring to greater longevity (because short telomeres are associated with earlier cellular senescence)^62^, which would be adaptive in an environment where reproduction is delayed. However, the greater intellectual and socioeconomic development in early adulthood of the offspring of older fathers, together with increased risk factors for later cardiovascular disease, rather suggests the opposite to this “slow” life history strategy.

### Strengths and weaknesses of the study

The Swedish national data cover almost the whole population, and the conscription medical examination data cover almost the whole male population, giving us confidence that our study sample is representative of the population. Furthermore, the size of this dataset gives us some power to distinguish correlated exposures such as maternal and paternal age while its historical depth allows us to examine the association of parental age with offspring adult outcomes, 18 years later. The study does, however, have limitations. We might hypothesise that the outcomes measured only in men at conscription medical examinations are similarly associated with parental age in women, but we have no evidence to test this except that those outcomes which were measured in both sexes showed reassuringly similar associations in sons and daughters with parental age.

The offspring in our study were born between 1951 and 1987. When the outcome of interest takes place 18 years after the exposure, it is inevitable that a study must be based on exposure data from the past. It is likely that the consequences of parental age for germline biology and the intrauterine environment are the same for children born today as for those born in the mid-late 20^th^ century. However, associations of parental age with offspring outcomes that are socioeconomically or behaviourally mediated might have changed due to changes in social structure or the division of childcare between mothers and fathers. This is an alternative explanation to the changing trajectory in parental age for some of the differences found when the periods before and after the end of 1969 were compared.

It was necessary to restrict participants in the study to those alive after 1^st^ July 1991 because of missingness among those who died before this date which was non-random with respect to their parents’ age. While avoiding a major source of bias, this does introduce the risk of a survivor bias if the association between parental age and outcomes among those who died before 1^st^ July 1991 was not representative of the association in the population as a whole. The exclusion of those with severe handicaps or missing parental data could have biased the results in a similar manner, although such exclusions were few. Inclusion of the potentially biased subset of offspring with non-missing data who died between 1961 and 30^th^ June 1991 (N = 56,901 sons) had a negligible effect on most results, but the sibling-comparison analyses of birth weight and birth length developed negative associations with maternal age (Supplementary Tables S14 and S15). This was probably due to a strong negative association between maternal age and birth weight and length among sons who died in infancy in this period. Sons included in the main analyses of birth weight and length had to survive to at least 3.5 years old. A similar potential bias exists for the other outcomes in the main analyses, most of which were necessarily restricted to sons who survived to adulthood. Hence, weaker offspring born to older mothers but who died in childhood might have been disproportionately excluded. Furthermore, mothers who were able to conceive at older ages might have been an unusually healthy subset of the population^12^.

The associations presented here only justify describing increased parental age as advantageous or disadvantageous if they are causal in origin. None of the methods we employ can claim to be an unbiased estimate of the direct causal effect of parental age on offspring outcomes. Instead, it is necessary to consider a number of methods, with their different weaknesses, in conjunction to make the best possible inferences regarding causal effects. We have taken measures to adjust for potential confounding factors, but strong socioeconomic patterning and secular trends in parental age and offspring outcomes make residual confounding possible. Because parental age is determined at the beginning of a child’s life, we consider most residual confounding likely to take place at the family level, making sibling-comparison analyses (which adjust for unmeasured confounding at this level) attractive. However, we also demonstrate that this method suffers from some severe limitations specific to the analysis of parental age, in particular the inability to adjust for within-family secular trends. Furthermore, adjustment for the other parent’s age at the offspring’s birth was possible in the sibling comparison analysis only because of those parents who had children with more than one partner. Otherwise, the ages at offspring birth of the two parents would have been perfectly correlated within families. Such families may not be typical representatives of the population in which to assess this important covariate, particularly because one biological parent will often not cohabit with their offspring. This will reduce the potential for that parent to influence offspring outcomes through behavioural or economic pathways.

In addition to the different unintended pathways associating outcome and exposure, both sibling comparison and two-variable methods exclude some categories of offspring. For the sibling comparison analysis these are sons who have no outcome-discordant brothers and for the two-variable analysis it is sons who are the oldest in their family. The remaining sample may not be typical of the population, especially given that only-children are among the exclusions from both analyses.

## Conclusions

The offspring of older mothers benefitted from a reduced tendency to smoke and from greater height, intelligence, non-cognitive ability and SEP but their increased adiposity and blood pressure probably puts them at greater risk of cardiovascular disease. Associations with paternal age were largely due to its association with maternal age, but associations in the same direction with greatly weakened magnitude remained after mutual adjustment. No single mechanism was adequate to account for the observed results, but they are consistent with older mothers, and perhaps fathers, having a greater ability to raise their offspring into intellectual and socioeconomic success, while also exposing them to an increased risk of hypertension and adiposity. Reduced birth weight in the offspring of older mothers suggests a poorer intrauterine environment, which may also be detrimental to adult cardiovascular health.

## Methods

### Selection of study subjects

The Swedish Multi-Generation Register 2013 includes 5,825,299 index persons (hereafter, offspring; sons and daughters) born between 1^st^ January 1932 and 31^st^ December 1987 and registered alive in Sweden in 1961 or later. Dates of birth (DOB) were provided already rounded to the nearest quarter-year, so this level of precision was used for all dates and ages. For many offspring who died between 1^st^ January 1969 and 30^th^ June 1991, the identity of their parents was missing and this missingness was not independent of the age of the parents. The data were thus limited to those offspring who were alive and living in Sweden on the 1^st^ July 1991 (N = 5,603,871). The data were restricted to those born from 1^st^ January 1951 onwards (N = 3,770,587) because most outcomes were unavailable for earlier cohorts. Offspring were also excluded if they were from multiple births (71,312 exclusions), or had an unidentified parent (45,337 exclusions). This gave a sample size of 1,873,803 sons and 1,780,135 daughters before outcome-specific exclusions. For the analysis of each outcome variable, offspring were excluded if they had missing data on that outcome.

### Data linkage

Data were linked to conscription medical examination records, providing additional data for male offspring only at a mean age of 18.3 (range 16.0 to 25.75, with 90.3% aged 17 or 18). Conscription examinations were compulsory for young Swedish men from 1969 until 2001 except for those with severe handicap or chronic disease. They provided data on height, BMI, systolic (SBP) and diastolic (DBP) blood pressure, intelligence, non-cognitive ability, handedness and smoking behavior. Details of the collection of these data are available in the supplement (Supplementary Methods). The Swedish Population and Housing Census provided data on educational and occupational socioeconomic position (SEP). Educational SEP was coded as seven levels according to the time spent in education and a binary outcome variable was created indicating whether or not the index person had completed secondary education. Occupational SEP was also coded into seven levels and a binary outcome variable was created combining the three non-manual categories. Further details are available in the supplement (Supplementary Methods). Linkage to the Medical Birth Register provided data on the birth weight and length of 98.3% of offspring born in 1973 or later. Linkage to the Swedish Cause of Death Register provided the date of death of those parents dying between 1^st^ January 1952 and 31^st^ March 2013. Birth order was coded according to the mother’s previous live births; none, one, two, or more than two. Variables for family size and birth order within the family were also created using only the offspring available in the full dataset, with families defined by maternal and paternal identity in turn. The study was approved (number 2016/5:5) by the Ethical Review Board in Stockholm, who operate according to Swedish national law and European guidelines and do not require informed consent for research based on non-identifiable register-based data.

### Descriptive statistics

Because most outcomes were only available for male offspring, analyses were conducted separately for sons and daughters. The average values in mothers, fathers and offspring of all measured variables potentially associated with parental age were calculated to describe the study population. The associations of paternal and maternal age with a suite of parental factors likely to influence them were analysed in unadjusted models with robust standard errors clustered by the parent’s identity. These factors were the offspring’s DOB and birth order, the age of the other parent at the offspring’s birth, and each parent’s occupational and educational SEP.

### Primary analyses

The primary statistical analyses consisted of regressions of offspring outcomes against each parent’s age at the time of the offspring’s birth. Continuous outcomes, analysed with linear regression, were: height; BMI; SBP; DBP; intelligence; non-cognitive ability; birth weight; and birth length. Binary outcomes, analysed with logistic regression, were: nonmanual employment; full secondary education; smoking; and being left-handed. A final binary outcome, offspring sex, was analysed using combined data for sons and daughters. Maternal and paternal age were analysed separately as the exposure of interest. Robust standard errors clustered by the identity of the parent in question were used to account for clustering within families. Linear (or logit-linear) models were used to represent associations across the whole range of parental age with a single, comparable statistic. To examine the shapes of the associations, the primary analyses were also conducted with parental age divided into categories, and the associations plotted. Categories of <20, 20–24, 25–29, 30–34, 35–39, 40–44 and ≥45 years were used for fathers, and for mothers the last two categories were combined due to the scarcity of mothers aged over 45.

### Adjustment

Analyses were conducted with six alternative adjustment sets. Adjustment (a) consisted of no adjustment; the age of the parent in question at offspring birth was the only covariate. Adjustment (b) consisted of the offspring’s DOB (as a cubic spline with knots at percentiles of 5%, 27.5%, 50%, 72.5% and 95%^63^, giving a flexible nonlinear fit). Adjustment (c) additionally adjusted for the occupational and educational SEP of both parents. Adjustment (d) further added the offspring’s birth order. Adjustment (e) was the default adjustment set for results given below. It consisted of adjustment (d), plus a linear term for the other parent’s age at the time of the offspring’s birth. Finally, adjustment (f) replaced the linear term for the other parent’s age with categories (as described below). When adjustment (e) was used, the resulting coefficients for maternal and paternal age were compared using a Wald test. When an adjustment variable was the outcome, it was removed from the adjustment set.

### Sibling-comparison analyses

We used a variety of secondary analyses, always with adjustment set (e), to investigate further the nature of the associations between parental age and the various outcomes. Sibling-comparison models allow within-family comparisons of outcomes against parental age, controlling for all unmeasured family-level factors^13^. To conduct sibling-comparison analyses, we applied fixed-effects linear regression to the continuous outcomes, and conditional logistic regression to the binary outcomes, with groups defined by the identity of the parent in question. Because offspring smoking status was recorded over such a short period, it was rarely available for a pair of siblings and was thus excluded as an outcome in these analyses. It was neither necessary nor possible to adjust for family-level covariates (the occupational and educational SEP of the parent in question), so these were omitted. A major disadvantage of sibling-comparison analyses of parental age is that while secular trends in the outcome at the family level are automatically adjusted for, no meaningful adjustment can be made for secular trends at the individual level^64^ (Supplementary Note online). This is because within families, parental age is perfectly correlated with offspring DOB. Offspring DOB was therefore omitted from the adjustment sets in the sibling-comparison analyses. Two tests were used to assess whether the results from the sibling-comparison analyses were due to secular trends. First, secular trends in offspring outcomes were quantified with the same data restrictions and adjustment sets as in the sibling-comparison analysis, but using linear or logistic regression with robust standard errors clustered by the parent’s identity. Second, the primary analyses were repeated with adjustment for the parent’s DOB instead of the offspring’s. By adjusting for secular trends at a family level, but not an individual level, this mimics the sibling comparison analysis except that it does not control for unmeasured family-level confounders (Supplementary Note online). Another disadvantage of sibling-comparison analyses is that the outcome-exposure association is influenced only by those offspring having at least one sibling who was available for use in the same analysis, and (for binary outcomes) had a different outcome value. The sibling comparison analysis was restricted to these individuals. To assess the effect of this restricted data set, the primary analyses, with parent’s DOB in place of offspring’s, were also conducted on the data subset used in the sibling-comparison analyses. Finally, the main sibling comparison analyses were repeated using categories of parental age instead of a single continuous exposure (as described for the primary analysis), and the results were plotted to examine the shapes of the associations.

### Two-variable analyses

An alternative method to assess the impact of family-level confounding is to restrict the analysis to those offspring who are not the oldest in the family, and include as covariates parental age (i) at the birth of the index offspring and (ii) at the birth of the oldest sibling^17^. In this mutually adjusted method, which we refer to hereafter as a “two-variable” analysis, an association with the parent’s age at the oldest sibling’s birth can be interpreted as a family-level effect probably due to unmeasured confounding, while an association with the parent’s age at the index child’s birth may be considered adjusted for such confounding, although there is a risk of induced confounding (Supplementary Note online). Family positions in this analysis were derived in terms of all male and female offspring of the parent in question who were included in the available data, and so did not always concur with the (maternal) birth order adjustment variable, which was therefore retained. To test whether associations between parental age and offspring outcomes differed for first-born and subsequent offspring (the former being omitted from the two-variable analysis), the primary analyses were repeated separately for first-born and later offspring. An analysis of first-born offspring also provides a strictly between-family analysis, and avoids any possible stoppage^65^ effects.

### Other sensitivity analyses

Mean parental age over the study period showed strong secular trends, and confounding by these trends is a major concern. We therefore repeated the primary and sibling-comparison analyses separately for those offspring born before or during 1969 (a period in which mean parental age was falling) and those born during or after 1970 (during which mean parental age was rising). Smoking behaviour, birth weight and birth length were omitted as outcomes because data were not available from both periods, and non-manual employment was omitted from the sibling-comparison analysis because few sibling pairs with this variable were available after 1969. Raw secular trends for parental age and for all outcomes were also plotted using the data used in the primary analyses.

To assess whether associations between the outcomes and parental age were mediated by intergenerational lifespan overlap^11^, the analyses were repeated on those offspring for whom it could be determined whether or not the parent in question lived until the offspring’s 16^th^ birthday. Parental survival follow-up was available from 1^st^ January 1952 until 31^st^ March 2013 and no date of death was available for those parents dying before this period. Therefore, parental survival to the offspring’s 16^th^ birthday could only be confidently determined for those offspring born from 1^st^ October 1952 (the parent must have been alive at the offspring’s conception, so could not have died before 1^st^ January 1952). Analyses restricted to those offspring with available data on parental survival to the offspring’s 16^th^ birthday were run with and without adjustment for this binary variable.

Sibling groups were defined in all main analyses by the identity of the “exposure” parent, meaning that they included half -siblings for whom the other parent was not the same. To examine the effect of this, the primary and sibling comparison analyses were re-run with sibling groups defined by the identities of both parents (half-siblings were thus treated as unrelated). In the sibling-comparison analysis, this meant that no parent-level covariates could be adjusted for.

Associations between parental age and offspring adult outcomes might be mediated by offspring birth weight and birth length. These variables were only available for offspring born from 1973 onwards (about 34% of all offspring). To assess the evidence for mediation, as well as any effects of this restriction of the data, the primary and sibling comparison analyses were repeated with and without adjustment for birth weight and birth length among those offspring for whom these variables were available. Because they were not available in offspring born after 1973, non-manual employment and smoking could not be analysed in this sensitivity analysis.

Analyses were conducted using Stata 12 on the University of Bristol supercomputer BlueCrystal.

## Additional Information

**How to cite this article:** Carslake, D. *et al*. Associations of parental age with health and social factors in adult offspring. Methodological pitfalls and possibilities. *Sci. Rep.* **7**, 45278; doi: 10.1038/srep45278 (2017).

**Publisher's note:** Springer Nature remains neutral with regard to jurisdictional claims in published maps and institutional affiliations.

## Supplementary Material

Supplementary Material

## Figures and Tables

**Table 1:** Description of the study sample.

Variable	N_primary_	Number of unique		Mean (SD) or Percentage	N_restricted_ (mothers)	N_restricted_ (fathers)
		Mothers	Fathers			
**Offspring:**						
Height at age 18 (cm)^a^	1,582,882	1,165,828	1,159,033	179.4 (6.5)	764,659	772,502
BMI at age 18 (kg m^−2^)^a^	1,582,530	1,165,665	1,158,866	21.9 (3.0)	764,329	772,187
SBP at age 18 (mmHg)^a^	1,507,349	1,116,992	1,110,961	128.6 (11.0)	716,047	723,080
DBP at age 18 (mmHg)^a^	1,507,140	1,116,910	1,110,865	67.6 (9.8)	715,833	722,898
Intelligence at age 18 (1–9)^a^	1,638,543	1,200,313	1,193,293	5.1 (1.9)	802,194	810,079
Non-cognitive ability at age 18 (1–9)^a^	1,075,579	834,128	829,482	5.2 (1.7)	447,037	453,620
Birth weight (hg)^a^	714,333	556,896	556,736	35.7 (5.5)	295,393	295,209
Birth length (cm)^a^	712,566	555,524	555,394	50.8 (2.3)	294,675	294,457
DOB (years)^a^	1,873,803	1,328,283	1,319,234	1969.1 (10.5)		
Non-manual employment^b^	845,979	671,301	668,332	37.5%	131,833	135,946
Full secondary education^b^	1,855,044	1,316,872	1,308,592	58.7%	344,373	357,344
Eldest child^b^	1,873,803	1,328,283	1,319,234	42.2%		
Smoker at age 18^b^	34,541	34,523	34,506	59.1%		
Left-handed^b^	1,300,097	974,408	969,884	8.4%	105,501	107,215
**Mother:**						
Age at offspring birth (years)^a^	1,873,803	1,328,283	1,319,234	27.6 (5.6)		
Non-manual employment^b^	1,644,018	1,163,526	1,163,138	50.7%		
Full secondary education^b^	1,525,968	1,061,477	1,063,754	24.3%		
Alive at offspring’s 16^th^ birthday^b^	1,823,990	1,295,883	1,287,093	98.9%		
Alive at offspring’s 40^th^ birthday^b^	1,114,037	824,060	819,914	88.3%		
**Father:**						
Age at offspring birth (years)^a^	1,873,803	1,328,283	1,319,234	30.7 (6.5)		
Non-manual employment^b^	1,351,336	970,440	950,511	68.2%		
Full secondary education^b^	1,578,517	1,105,260	1,087,605	33.6%		
Alive at offspring’s 16^th^ birthday^b^	1,823,968	1,295,865	1,287,071	97.3%		
Alive at offspring’s 40^th^ birthday^b^	1,114,015	824,042	819,892	74.0%		

Means and percentages are calculated from the data available for the primary analysis. For those outcomes included in the sibling comparison analysis, sample sizes are also given for the restricted data (sons having a brother with a discordant outcome value). ^a^Continuous variables are described as means (SD). ^b^Binary variables are described as percentages.

**Table 2:** Associations between son outcomes and maternal age from primary and sibling comparison analyses.

Outcome	Association per five years of mother’s age at son’s birth (or per five years of son’s DOB for secular trend)				
	Primary analysis	Primary Analysis (mother’s DOB)	Primary Analysis (mother’s DOB, restricted data)	Sibling-comparison analysis (restricted data)	Secular trend (restricted data)
Height at 18 (cm)^a^	0.42 (0.40, 0.44)	0.61 (0.59, 0.63)	0.68 (0.66, 0.71)	0.69 (0.64, 0.74)	0.25 (0.24, 0.26)
BMI at 18 (kg m^−2^)^a^	0.00 (−0.01, 0.01)	0.29 (0.28, 0.30)	0.30 (0.29, 0.31)	0.59 (0.56, 0.62)	0.29 (0.28, 0.29)
SBP at 18 (mmHg)^a^	0.39 (0.36, 0.42)	0.80 (0.77, 0.83)	0.74 (0.69, 0.79)	0.70 (0.59, 0.82)	0.24 (0.22, 0.25)
DBP at 18 (mmHg)^a^	0.13 (0.11, 0.15)	−0.44 (−0.47, −0.42)	−0.52 (−0.56, −0.48)	−0.19 (−0.29, −0.09)	−0.68 (−0.70, −0.67)
Intelligence at 18 (1–9)^a^	0.19 (0.18, 0.19)	0.09 (0.08, 0.09)	0.12 (0.12, 0.13)	0.07 (0.05, 0.08)	−0.05 (−0.06, −0.05)
Non-cognitive ability at 18 (1–9)^a^	0.08 (0.07, 0.08)	0.09 (0.09, 0.10)	0.14 (0.13, 0.15)	0.08 (0.05, 0.10)	0.08 (0.08, 0.09)
Birth weight (hg)^a^	−0.14 (−0.16, −0.11)	0.00 (−0.02, 0.03)	0.11 (0.07, 0.16)	−0.01 (−0.11, 0.08)	0.15 (0.11, 0.18)
Birth length (cm)^a^	−0.02 (−0.03, −0.01)	−0.04 (−0.05, −0.03)	−0.01 (−0.03, 0.01)	−0.03 (−0.07, 0.02)	−0.02 (−0.03, 0.00)
Non-manual employment^b^	1.21 (1.21, 1.22)	0.63 (0.62, 0.63)	0.71 (0.70, 0.72)	0.63 (0.60, 0.66)	0.79 (0.78, 0.79)
Full secondary education^b^	1.31 (1.31, 1.32)	1.74 (1.73, 1.75)	1.37 (1.35, 1.38)	1.86 (1.82, 1.91)	1.10 (1.10, 1.10)
Smoker at 18^b^	0.86 (0.83, 0.88)	0.68 (0.48, 0.95)			
Left-handed^b^	0.97 (0.96, 0.98)	0.95 (0.94, 0.96)	1.03 (1.01, 1.04)	0.90 (0.86, 0.95)	1.02 (1.01, 1.02)

Primary analyses and the secular trends analyses used linear or logistic regression with robust standard errors clustered by maternal identity. The sibling-comparison analysis used fixed-effects linear or conditional logistic regression grouped by maternal identity. Adjustment set (e) (offspring DOB, maternal and paternal occupational and educational SEP, offspring birth order, and paternal age) was used for all analyses, except that offspring DOB was replaced by maternal DOB where indicated and that mother-level terms and offspring DOB were omitted in the sibling-comparison analysis. Restricted data, a necessity for the sibling comparison analysis, consisted of those sons who had a brother in the dataset. ^a^Continuous outcomes; associations are mean differences (95% CI). ^b^Binary outcomes; associations are odds ratios (95% CI).

**Table 3:** Associations between son outcomes and paternal age from primary and sibling comparison analyses.

Outcome	Association per five years of father’s age at son’s birth (or per five years of son’s DOB for secular trend)				
	Primary analysis	Primary Analysis (father’s DOB)	Primary Analysis (father’s DOB, restricted data)	Sibling-comparison analysis (restricted data)	Secular trend (restricted data)
Height at 18 (cm)^a^	−0.02 (−0.03, −0.01)	0.16 (0.14, 0.17)	0.17 (0.14, 0.19)	0.39 (0.35, 0.43)	0.16 (0.15, 0.17)
BMI at 18 (kg m^−2^)^a^	0.01 (0.00, 0.02)	0.30 (0.29, 0.31)	0.32 (0.31, 0.33)	0.40 (0.38, 0.42)	0.29 (0.28, 0.29)
SBP at 18 (mmHg)^a^	0.10 (0.07, 0.12)	0.50 (0.48, 0.53)	0.43 (0.39, 0.47)	0.63 (0.54, 0.72)	0.27 (0.25, 0.29)
DBP at 18 (mmHg)^a^	0.04 (0.02, 0.06)	−0.76 (−0.78, −0.74)	−0.75 (−0.78, −0.71)	−0.42 (−0.50, −0.34)	−0.77 (−0.78, −0.75)
Intelligence at 18 (1–9)^a^	0.03 (0.02, 0.03)	−0.07 (−0.08, −0.07)	−0.08 (−0.08, −0.07)	−0.07 (−0.09, −0.06)	−0.12 (−0.12, −0.12)
Non-cognitive ability at 18 (1–9)^a^	−0.01 (−0.02, −0.01)	0.00 (0.00, 0.01)	0.03 (0.02, 0.04)	0.12 (0.10, 0.14)	0.06 (0.05, 0.06)
Birth weight (hg)^a^	−0.04 (−0.06, −0.03)	0.09 (0.07, 0.12)	0.12 (0.08, 0.17)	0.19 (0.10, 0.29)	0.11 (0.07, 0.15)
Birth length (cm)^a^	−0.02 (−0.03, −0.02)	−0.05 (−0.06, −0.03)	−0.04 (−0.06, −0.02)	−0.02 (−0.07, 0.02)	−0.04 (−0.06, −0.02)
Non-manual employment^b^	1.01 (1.00, 1.02)	0.54 (0.53, 0.54)	0.70 (0.69, 0.71)	0.59 (0.57, 0.62)	0.76 (0.76, 0.77)
Full secondary education^b^	1.04 (1.04, 1.05)	1.35 (1.35, 1.36)	1.26 (1.25, 1.27)	1.44 (1.41, 1.47)	1.10 (1.10, 1.10)
Smoker at 18^b^	0.91 (0.89, 0.94)	0.74 (0.53, 1.04)			
Left-handed^b^	1.00 (0.99, 1.00)	0.98 (0.97, 0.99)	0.97 (0.96, 0.98)	0.93 (0.90, 0.97)	1.01 (1.00, 1.01)

Primary analyses and the secular trends analyses used linear or logistic regression with robust standard errors clustered by paternal identity. The sibling-comparison analysis used fixed-effects linear or conditional logistic regression grouped by paternal identity. Adjustment set (e) (offspring DOB, maternal and paternal occupational and educational SEP, offspring birth order, and maternal age) was used for all analyses, except that offspring DOB was replaced by paternal DOB where indicated and that father-level terms and offspring DOB were omitted in the sibling-comparison analysis. Restricted data, a necessity for the sibling comparison analysis, consisted of those sons who had a brother in the dataset. ^a^Continuous outcomes; associations are mean differences (95% CI). ^b^Binary outcomes; associations are odds ratios (95% CI).
