# Supplementary material for: Associations of parental age with health and social factors in adult offspring. Methodological pitfalls and possibilities
Source: Sci Rep. 2017 Mar 27;7:45278. doi: 10.1038/srep45278 (PMC5366914; doi:10.1038/srep45278)
Supplement: Supplementary Material [file srep45278-s1.pdf]

Supplementary material

Associations of parental age with health and social factors in adult offspring. Methodological pitfalls and possibilities

David Carslake[1,2]*, Per Tynelius[3], Gerard van den Berg[4], George Davey Smith[1,2] and Finn Rasmussen[3]

[1]MRC Integrative Epidemiology Unit at the University of Bristol, Bristol, UK, [2]School of Social and Community Medicine, University of Bristol, Bristol, UK, [3]Department of Public Health Sciences, Karolinska Institute, Stockholm, Sweden, [4]School of Economics, Finance and Management, University of Bristol, Bristol, UK.
*Correspondence and requests for materials should be addressed to D. C. (email: David.Carslake@bristol.ac.uk)

Supplementary Table S1. Description of the study population (daughters).
Supplementary Table S2. Associations between daughter outcomes and maternal age from primary and sibling comparison analyses.
Supplementary Table S3. Associations between daughter outcomes and paternal age from primary and sibling comparison analyses.
Supplementary Table S4. Primary analyses of son outcomes and maternal age with alternative adjustment sets.
Supplementary Table S5. Primary analyses of son outcomes and paternal age with alternative adjustment sets.
Supplementary Table S6. Analyses of son outcomes and maternal age, conducted separately for sons who were the oldest child in their family and for others. Two-variable analyses of later children were also conducted with mutual adjustment for mother's age at the birth of (i) the child in question, and (ii) her first child.
Supplementary Table S7. Analyses of son outcomes and paternal age, conducted separately for sons who were the oldest child in their family and for others. Two-variable analyses of later children were also conducted with mutual adjustment for father's age at the birth of (i) the child in question, and (ii) his first child.
Supplementary Table S8. Primary analyses of son outcomes and maternal age, conducted separately for sons born up to 1969 (when parental age was decreasing) and for sons born from 1970 (when parental age was increasing).
Supplementary Table S9. Primary analyses of son outcomes and paternal age, conducted separately for sons born up to 1969 (when parental age was decreasing) and for sons born from 1970 (when parental age was increasing).
Supplementary Table S10. Sibling comparison analyses of son outcomes and maternal age, conducted separately for sons born up to 1969 (when parental age was decreasing) and for sons born from 1970 (when parental age was increasing).
Supplementary Table S11. Sibling comparison analyses of son outcomes and paternal age, conducted separately for sons born up to 1969 (when parental age was decreasing) and for sons born from 1970 (when parental age was increasing).
Supplementary Table S12. Analyses of son's outcomes and maternal age, with and without additional adjustment for maternal survival to the offspring's 16th birthday.

Supplementary Table S13. Analyses of son's outcomes and paternal age, with and without additional adjustment for paternal survival to the offspring's 16th birthday.
Supplementary Table S14. Primary analyses of son outcomes and maternal age, with and without additional adjustment for offspring birth weight and birth length.
Supplementary Table S15. Primary analyses of son outcomes and paternal age, with and without additional adjustment for offspring birth weight and birth length.
Supplementary Table S16. Sibling comparison analyses of son outcomes and maternal age, with and without additional adjustment for offspring birth weight and birth length.
Supplementary Table S17. Sibling comparison analyses of son outcomes and paternal age, with and without additional adjustment for offspring birth weight and birth length.
Supplementary Table S18. Associations between son outcomes and maternal age from primary and sibling comparison analyses with families defined by both parents' identities.
Supplementary Table S19. Associations between son outcomes and paternal age from primary and sibling comparison analyses with families defined by both parents' identities.
Supplementary Table S20. Associations between son outcomes and maternal age from primary and sibling comparison analyses, including the potentially biased subset of sons who died between 1961 and 30th June 1991 (N=56,901).
Supplementary Table S21. Associations between son outcomes and paternal age from primary and sibling comparison analyses, including the potentially biased subset of sons who died between 1961 and 30th June 1991 (N=56,901).

Supplementary Figure S1. Unadjusted associations of paternal (filled circles) and maternal (open circles) age with factors among the parents of sons.
Supplementary Figure S2. Trends in parental age and outcome variables over the course of the study. 95% confidence intervals are shown where they exceed 10% of the plotted range. Trends for sons (black circles), daughters (white circles) or all offspring (grey circles) are plotted for three-year intervals of offspring date of birth, except for smoking, which is plotted for each year in which it was recorded. Trends for parental age and survival were indistinguishable for sons and daughters (data not shown), so were plotted for all offspring.

Supplementary Methods. Additional details of data preparation.

Supplementary Note. Secular trends in sibling-comparison analyses of parental age. A simulation study of methodology.

Supplementary Table S1. Description of the study sample (daughters). [a]Continuous variables are described as means (SD). [b]Binary variables are described as percentages.

Variable	$N_{primary}$	Number of unique Mothers	Number of unique Fathers	Mean (SD) or Percentage
**Offspring:**				
Birth weight (hg)[a]	676,709	534,955	534,846	34.4 (5.2)
Birth length (cm)[a]	675,182	533,826	533,724	50.0 (2.2)
DOB (years)[a]	1,780,135	1,284,031	1,274,883	1969.1 (10.5)
Non-manual employment[b]	931,009	725,249	722,310	42.4%
Full secondary education[b]	1,762,003	1,272,768	1,264,434	68.9%
Male sex[b]	1,780,135	1,284,031	1,274,883	0.0%
Eldest child[b]	1,780,135	1,284,031	1,274,883	42.2%
**Mother:**				
Age at offspring birth (years)[a]	1,780,135	1,284,031	1,274,883	27.6 (5.6)
Non-manual employment[b]	1,559,672	1,123,849	1,123,121	50.7%
Full secondary education[b]	1,450,573	1,028,230	1,029,829	24.1%
Alive at offspring's 16th birthday[b]	1,732,367	1,252,334	1,243,547	98.9%
Alive at offspring's 40th birthday[b]	1,059,809	795,064	791,176	88.3%
**Father:**				
Age at offspring birth (years)[a]	1,780,135	1,284,031	1,274,883	30.7 (6.5)
Non-manual employment[b]	1,281,948	936,761	917,928	68.1%
Full secondary education[b]	1,499,438	1,069,683	1,052,788	33.4%
Alive at offspring's 16th birthday[b]	1,732,356	1,252,324	1,243,536	97.2%
Alive at offspring's 40th birthday[b]	1,059,798	795,054	791,165	74.0%

Supplementary Table S2. Associations between daughter outcomes and maternal age from primary and sibling comparison analyses. Primary analyses and the secular trends analyses used linear or logistic regression with robust standard errors clustered by maternal identity. The sibling-comparison analysis used fixed-effects linear or conditional logistic regression grouped by maternal identity. Adjustment set (e) (offspring DOB, maternal and paternal occupational and educational SEP, offspring birth order, and paternal age) was used for all analyses, except that offspring DOB was replaced by maternal DOB where indicated and that mother-level terms and offspring DOB were omitted in the sibling-comparison analysis. Restricted data, a necessity for the sibling comparison analysis, consisted of those daughters who had a sister in the dataset. Offspring of both sexes were used for the analysis of sex. [a]Continuous outcomes. Associations are mean differences (95% CI). [b]Binary outcomes. Associations are odds ratios (95% CI).

	**Association per five years of mother's age at daughter's birth (or per five years of daughter's DOB for secular trend)**				
**Outcome**	**Primary analysis**	**Primary Analysis (mother's DOB)**	**Primary Analysis (mother's DOB, restricted data)**	**Sibling-comparison analysis (restricted data)**	**Secular trend (restricted data)**
Birth weight (hg)[a]	-0.12 (-0.15, -0.10)	0.01 (-0.02, 0.03)	0.15 (0.10, 0.20)	0.08 (-0.01, 0.18)	0.16 (0.13, 0.20)
Birth length (cm)[a]	-0.02 (-0.03, -0.01)	-0.06 (-0.07, -0.04)	0.00 (-0.02, 0.02)	0.01 (-0.03, 0.05)	-0.02 (-0.03, 0.00)
Non-manual employment[b]	1.25 (1.24, 1.26)	0.74 (0.73, 0.74)	0.80 (0.79, 0.81)	0.75 (0.72, 0.78)	0.84 (0.84, 0.85)
Full secondary education[b]	1.30 (1.30, 1.31)	1.79 (1.77, 1.80)	1.37 (1.36, 1.38)	1.83 (1.79, 1.88)	1.11 (1.11, 1.12)
Male sex[b]	1.00 (1.00, 1.01)	1.00 (1.00, 1.01)	1.00 (1.00, 1.00)	1.01 (1.00, 1.02)	1.00 (1.00, 1.00)

Supplementary Table S3. Associations between daughter outcomes and paternal age from primary and sibling comparison analyses. Primary analyses and the secular trends analyses used linear or logistic regression with robust standard errors clustered by paternal identity. The sibling-comparison analysis used fixed-effects linear or conditional logistic regression grouped by paternal identity. Adjustment set (e) (offspring DOB, maternal and paternal occupational and educational SEP, offspring birth order, and paternal age) was used for all analyses, except that offspring DOB was replaced by paternal DOB where indicated and that father-level terms and offspring DOB were omitted in the sibling-comparison analysis. Restricted data, a necessity for the sibling comparison analysis, consisted of those daughters who had a sister in the dataset. Offspring of both sexes were used for the analysis of sex. [a]Continuous outcomes. Associations are mean differences (95% CI). [b]Binary outcomes. Associations are odds ratios (95% CI).

	**Association per five years of father's age at daughter's birth (or per five years of daughter's DOB for secular trend)**				
**Outcome**	**Primary analysis**	**Primary Analysis (father's DOB)**	**Primary Analysis (father's DOB, restricted data)**	**Sibling-comparison analysis (restricted data)**	**Secular trend (restricted data)**
Birth weight (hg)[a]	-0.04 (-0.05, -0.02)	0.08 (0.06, 0.11)	0.07 (0.02, 0.12)	0.14 (0.05, 0.24)	0.08 (0.04, 0.11)
Birth length (cm)[a]	-0.02 (-0.03, -0.02)	-0.07 (-0.08, -0.05)	-0.06 (-0.09, -0.04)	-0.05 (-0.09, 0.00)	-0.05 (-0.07, -0.04)
Non-manual employment[b]	1.02 (1.01, 1.02)	0.62 (0.61, 0.62)	0.76 (0.75, 0.77)	0.63 (0.61, 0.66)	0.82 (0.81, 0.82)
Full secondary education[b]	1.05 (1.04, 1.05)	1.43 (1.42, 1.43)	1.29 (1.28, 1.30)	1.46 (1.43, 1.49)	1.12 (1.12, 1.13)
Male sex[b]	1.00 (1.00, 1.00)	1.00 (1.00, 1.00)	1.00 (1.00, 1.01)	1.01 (1.00, 1.02)	1.00 (1.00, 1.00)

Supplementary Table S4. Primary analyses of son outcomes and maternal age with alternative adjustment sets. Adjustment set (a) was empty, (b) comprised a cubic spline of offspring DOB, (c) additionally included SEP in both parents and (d) additionally included birth order. Adjustment set (e) additionally included paternal age as a linear term. Adjustment variables were excluded when they matched the outcome. Sample sizes were as described in Table 1. Maternal and paternal associations in model (e) were compared using P-values from a Wald test. [a]Continuous outcomes. Associations are mean differences (95% CI) from linear regression with robust standard errors clustered by maternal identity. [b]Binary outcomes. Associations are odds ratios (95% CI) from logistic regression with robust standard errors clustered by maternal identity.

	**Primary association per five years of mother's age at son's birth with adjustment:**					**Parental**
**Outcome**	**a**	**b**	**c**	**d**	**e**	**comparison**
Height at 18 (cm)[a]	0.28 (0.27, 0.28)	0.29 (0.28, 0.30)	0.24 (0.23, 0.25)	0.41 (0.39, 0.42)	0.42 (0.40, 0.44)	<0.001
BMI at 18 (kg m^{-2})[a]	-0.04 (-0.04, -0.03)	-0.03 (-0.04, -0.03)	0.00 (-0.01, 0.00)	0.01 (0.00, 0.01)	0.00 (-0.01, 0.01)	0.135
SBP at 18 (mmHg)[a]	0.30 (0.28, 0.31)	0.31 (0.29, 0.33)	0.26 (0.25, 0.28)	0.47 (0.45, 0.49)	0.39 (0.36, 0.42)	<0.001
DBP at 18 (mmHg)[a]	0.40 (0.39, 0.41)	0.09 (0.07, 0.10)	0.10 (0.09, 0.12)	0.16 (0.15, 0.18)	0.13 (0.11, 0.15)	<0.001
Intelligence at 18 (1-9)[a]	0.15 (0.15, 0.16)	0.16 (0.15, 0.16)	0.09 (0.09, 0.10)	0.21 (0.21, 0.21)	0.19 (0.18, 0.19)	<0.001
Non-cognitive ability at 18 (1-9)[a]	-0.01 (-0.01, 0.00)	0.01 (0.01, 0.01)	0.01 (0.01, 0.01)	0.07 (0.06, 0.07)	0.08 (0.07, 0.08)	<0.001
Birth weight (hg)[e]	0.33 (0.31, 0.34)	0.32 (0.31, 0.34)	0.19 (0.17, 0.21)	-0.17 (-0.19, -0.15)	-0.14 (-0.16, -0.11)	<0.001
Birth length (cm)[a]	0.09 (0.09, 0.10)	0.10 (0.10, 0.11)	0.04 (0.03, 0.05)	-0.04 (-0.05, -0.03)	-0.02 (-0.03, -0.01)	0.790
Non-manual employment[b]	1.14 (1.14, 1.15)	1.07 (1.07, 1.08)	1.13 (1.12, 1.13)	1.23 (1.22, 1.23)	1.21 (1.21, 1.22)	<0.001
Full secondary education[b]	1.19 (1.19, 1.20)	1.20 (1.19, 1.20)	1.18 (1.18, 1.19)	1.36 (1.36, 1.37)	1.31 (1.31, 1.32)	<0.001
Smoker at 18[b]	0.91 (0.89, 0.93)	0.91 (0.89, 0.92)	0.90 (0.88, 0.92)	0.80 (0.79, 0.82)	0.86 (0.83, 0.88)	0.014
Left-handed[b]	0.99 (0.98, 0.99)	0.99 (0.98, 0.99)	0.99 (0.98, 1.00)	0.97 (0.96, 0.97)	0.97 (0.96, 0.98)	<0.001

Supplementary Table S5. Primary analyses of son outcomes and paternal age with alternative adjustment sets. Adjustment set (a) was empty, (b) comprised a cubic spline of offspring DOB, (c) additionally included SEP in both parents and (d) additionally included birth order. Adjustment set (e) additionally included maternal age as a linear term. Adjustment variables were excluded when they matched the outcome. Sample sizes were as described in Table 1. Maternal and paternal associations in model (e) were compared using P-values from a Wald test. [a]Continuous outcomes. Associations are mean differences (95% CI) from linear regression with robust standard errors clustered by paternal identity. [b]Binary outcomes. Associations are odds ratios (95% CI) from logistic regression with robust standard errors clustered by paternal identity.

	**Primary association per five years of father's age at son's birth with adjustment:**					**Parental comparison**
**Outcome**	**a**	**b**	**c**	**d**	**e**	
Height at 18 (cm)[a]	0.11 (0.10, 0.12)	0.13 (0.13, 0.14)	0.13 (0.12, 0.14)	0.21 (0.20, 0.22)	-0.02 (-0.03, -0.01)	<0.001
BMI at 18 (kg m^{-2})[a]	-0.02 (-0.03, -0.02)	0.00 (-0.01, 0.00)	0.00 (0.00, 0.01)	0.01 (0.01, 0.01)	0.01 (0.00, 0.02)	0.135
SBP at 18 (mmHg)[a]	0.22 (0.20, 0.23)	0.24 (0.23, 0.25)	0.19 (0.18, 0.21)	0.31 (0.29, 0.33)	0.10 (0.07, 0.12)	<0.001
DBP at 18 (mmHg)[a]	0.34 (0.33, 0.35)	0.06 (0.05, 0.08)	0.08 (0.06, 0.09)	0.11 (0.10, 0.13)	0.04 (0.02, 0.06)	<0.001
Intelligence at 18 (1-9)[a]	0.07 (0.07, 0.08)	0.07 (0.07, 0.08)	0.06 (0.06, 0.06)	0.13 (0.13, 0.13)	0.03 (0.02, 0.03)	<0.001
Non-cognitive ability at 18 (1-9)[a]	-0.04 (-0.05, -0.04)	-0.03 (-0.03, -0.02)	0.00 (-0.01, 0.00)	0.03 (0.03, 0.04)	-0.01 (-0.02, -0.01)	<0.001
Birth weight (hg)[e]	0.22 (0.20, 0.23)	0.21 (0.20, 0.23)	0.08 (0.06, 0.09)	-0.10 (-0.12, -0.09)	-0.04 (-0.06, -0.03)	<0.001
Birth length (cm)[a]	0.05 (0.05, 0.06)	0.06 (0.06, 0.07)	0.01 (0.00, 0.01)	-0.03 (-0.04, -0.03)	-0.02 (-0.03, -0.02)	0.790
Non-manual employment[b]	1.08 (1.08, 1.08)	1.01 (1.01, 1.01)	1.08 (1.08, 1.08)	1.13 (1.13, 1.14)	1.01 (1.00, 1.02)	<0.001
Full secondary education[b]	1.07 (1.06, 1.07)	1.09 (1.09, 1.09)	1.12 (1.12, 1.12)	1.21 (1.21, 1.22)	1.04 (1.04, 1.05)	<0.001
Smoker at 18[b]	0.93 (0.92, 0.95)	0.93 (0.92, 0.95)	0.91 (0.89, 0.93)	0.84 (0.82, 0.85)	0.91 (0.89, 0.94)	0.014
Left-handed[b]	1.00 (0.99, 1.00)	0.99 (0.99, 1.00)	0.99 (0.99, 1.00)	0.98 (0.97, 0.99)	1.00 (0.99, 1.00)	<0.001

Supplementary Table S6. Analyses of son outcomes and maternal age, conducted separately for sons who were the oldest child in their family and for others. Two-variable analyses of later children were also conducted with mutual adjustment for mother's age at the birth of (i) the child in question, and (ii) her first child. [a]Continuous outcomes. Associations are mean differences (95% CI) from linear regression with robust standard errors clustered by maternal identity. [b]Binary outcomes. Associations are odds ratios (95% CI) from logistic regression with robust standard errors clustered by maternal identity. Adjustment set (e) was used; a cubic spline of offspring DOB, SEP in both parents, birth order and paternal age at son's birth.

	Sample size (mothers)		Primary association per five years of mother's age at son's birth among:		Two-variable association among later sons, per five years of mother's age at	
**Outcome**	**Oldest offspring**	**Later offspring**	**Oldest offspring**	**Later offspring**	**Son's birth**	**Oldest offspring birth**
Height at 18 (cm)[a]	683,052	899,830 (748,745)	0.39 (0.37, 0.42)	0.45 (0.43, 0.47)	0.34 (0.32, 0.37)	0.17 (0.14, 0.20)
BMI at 18 (kg m^{-2})[a]	682,895	899,635 (748,623)	-0.02 (-0.03, -0.01)	0.01 (0.00, 0.02)	0.19 (0.18, 0.20)	-0.28 (-0.29, -0.27)
SBP at 18 (mmHg)[a]	649,886	857,463 (714,888)	0.49 (0.44, 0.53)	0.32 (0.29, 0.36)	0.30 (0.26, 0.35)	0.03 (-0.02, 0.08)
DBP at 18 (mmHg)[a]	649,803	857,337 (714,818)	0.16 (0.12, 0.20)	0.11 (0.08, 0.14)	0.26 (0.22, 0.30)	-0.24 (-0.28, -0.20)
Intelligence at 18 (1-9)[a]	705,431	933,112 (773,577)	0.21 (0.20, 0.21)	0.18 (0.17, 0.18)	0.09 (0.09, 0.10)	0.13 (0.13, 0.14)
Non-cognitive ability at 18 (1-9)[a]	471,685	603,894 (513,591)	0.06 (0.06, 0.07)	0.09 (0.08, 0.10)	0.08 (0.07, 0.09)	0.01 (0.01, 0.02)
Birth weight (hg)[e]	308,529	405,804 (354,981)	-0.15 (-0.18, -0.11)	-0.13 (-0.16, -0.09)	-0.39 (-0.43, -0.35)	0.44 (0.40, 0.47)
Birth length (cm)[a]	307,529	405,037 (354,348)	-0.03 (-0.04, -0.01)	-0.02 (-0.03, -0.01)	-0.11 (-0.13, -0.09)	0.15 (0.14, 0.17)
Non-manual employment[b]	371,874	474,105 (407,501)	1.21 (1.20, 1.23)	1.22 (1.21, 1.23)	1.23 (1.21, 1.24)	0.99 (0.98, 1.00)
Full secondary education[b]	798,048	1,056,996 (858,635)	1.36 (1.35, 1.37)	1.29 (1.28, 1.29)	1.15 (1.14, 1.16)	1.19 (1.18, 1.21)
Smoker at 18[b]	13,705	20,836 (20,828)	0.83 (0.79, 0.87)	0.88 (0.84, 0.91)	0.98 (0.93, 1.03)	0.85 (0.81, 0.90)
Left-handed[b]	562,138	737,959 (617,638)	0.99 (0.97, 1.00)	0.96 (0.95, 0.97)	0.95 (0.93, 0.96)	1.02 (1.00, 1.04)

Supplementary Table S7. Analyses of son outcomes and paternal age, conducted separately for sons who were the oldest child in their family and for others. Two-variable analyses of later children were also conducted with mutual adjustment for father's age at the birth of (i) the child in question, and (ii) his first child. [a]Continuous outcomes. Associations are mean differences (95% CI) from linear regression with robust standard errors clustered by paternal identity. [b]Binary outcomes. Associations are odds ratios (95% CI) from logistic regression with robust standard errors clustered by paternal identity. Adjustment set (e) was used; a cubic spline of offspring DOB, SEP in both parents, birth order and maternal age at son's birth.

	Sample size (fathers)		Primary association per five years of father's age at son's birth among:		Two-variable association among later sons, per five years of father's age at	
**Outcome**	**Oldest offspring**	**Later offspring**	**Oldest offspring**	**Later offspring**	**Son's birth**	**Oldest offspring birth**
Height at 18 (cm)[a]	690,526	892,356 (734,060)	-0.02 (-0.04, 0.00)	-0.02 (-0.04, -0.01)	-0.09 (-0.12, -0.07)	0.11 (0.09, 0.13)
BMI at 18 (kg m^{-2})[a]	690,370	892,160 (733,937)	0.01 (0.00, 0.02)	0.02 (0.01, 0.03)	0.11 (0.09, 0.12)	-0.14 (-0.15, -0.12)
SBP at 18 (mmHg)[a]	657,754	849,595 (700,864)	0.18 (0.14, 0.21)	0.08 (0.05, 0.11)	0.02 (-0.02, 0.06)	0.09 (0.05, 0.13)
DBP at 18 (mmHg)[a]	657,679	849,461 (700,778)	0.06 (0.03, 0.09)	0.04 (0.02, 0.07)	0.13 (0.10, 0.17)	-0.14 (-0.17, -0.11)
Intelligence at 18 (1-9)[a]	713,331	925,212 (758,152)	0.05 (0.05, 0.06)	0.03 (0.03, 0.03)	-0.04 (-0.05, -0.04)	0.11 (0.11, 0.12)
Non-cognitive ability at 18 (1-9)[a]	476,825	598,754 (504,931)	-0.01 (-0.02, -0.01)	-0.01 (-0.01, 0.00)	-0.02 (-0.02, -0.01)	0.01 (0.00, 0.02)
Birth weight (hg)[e]	309,462	404,871 (350,865)	-0.02 (-0.05, 0.01)	-0.04 (-0.06, -0.02)	-0.17 (-0.19, -0.14)	0.25 (0.23, 0.28)
Birth length (cm)[a]	308,477	404,089 (350,271)	-0.02 (-0.03, 0.00)	-0.02 (-0.03, -0.01)	-0.06 (-0.07, -0.05)	0.09 (0.07, 0.10)
Non-manual employment[b]	376,506	469,473 (400,643)	1.01 (1.01, 1.02)	1.01 (1.01, 1.02)	1.03 (1.01, 1.04)	0.99 (0.98, 1.00)
Full secondary education[b]	806,875	1,048,169 (840,898)	1.09 (1.08, 1.10)	1.04 (1.03, 1.04)	0.93 (0.93, 0.94)	1.17 (1.16, 1.18)
Smoker at 18[b]	14,481	20,060 (20,044)	0.90 (0.87, 0.94)	0.92 (0.89, 0.95)	1.02 (0.96, 1.08)	0.89 (0.84, 0.94)
Left-handed[b]	570,293	729,804 (605,119)	1.01 (0.99, 1.02)	0.99 (0.98, 1.00)	0.98 (0.97, 1.00)	1.01 (0.99, 1.02)

Supplementary Table S8. Primary analyses of son outcomes and maternal age, conducted separately for sons born up to 1969 (when parental age was decreasing) and for sons born from 1970 (when parental age was increasing). [a]Continuous outcomes. Associations are mean differences (95% CI) from linear regression with robust standard errors clustered by maternal identity. [b]Binary outcomes. Associations are odds ratios (95% CI) from logistic regression with robust standard errors clustered by maternal identity. Adjustment set (e) was used; a cubic spline of offspring DOB, SEP in both parents, birth order, and paternal age at son's birth. Data on birth weight, birth length and smoking status were only available in one period and were thus omitted as outcomes.

	**Sample size (mothers)**		**Primary association per five years of mother's age at son's birth**	
**Outcome**	**DOB<=1969**	**DOB>=1970**	**DOB<=1969**	**DOB>=1970**
Height at 18 (cm)[a]	884,453 (679,853)	698,429 (556,368)	0.45 (0.42, 0.47)	0.38 (0.35, 0.41)
BMI at 18 (kg m^{-2})[a]	884,127 (679,685)	698,403 (556,352)	0.02 (0.01, 0.03)	-0.03 (-0.04, -0.01)
SBP at 18 (mmHg)[a]	884,385 (679,814)	622,964 (505,411)	0.35 (0.32, 0.39)	0.38 (0.33, 0.43)
DBP at 18 (mmHg)[a]	884,320 (679,781)	622,820 (505,321)	0.10 (0.08, 0.13)	0.21 (0.16, 0.25)
Intelligence at 18 (1-9)[a]	893,160 (685,229)	745,383 (586,768)	0.20 (0.19, 0.21)	0.17 (0.17, 0.18)
Non-cognitive ability at 18 (1-9)[a]	812,510 (634,098)	263,069 (233,734)	0.09 (0.08, 0.09)	0.03 (0.02, 0.04)
Non-manual employment[b]	721,947 (577,750)	124,032 (120,853)	1.22 (1.21, 1.23)	1.08 (1.04, 1.12)
Full secondary education[b]	981,008 (731,617)	874,036 (666,532)	1.31 (1.30, 1.32)	1.32 (1.31, 1.33)
Left-handed[b]	861,342 (666,516)	438,755 (372,607)	0.96 (0.95, 0.97)	0.99 (0.97, 1.00)

Supplementary Table S9. Primary analyses of son outcomes and paternal age, conducted separately for sons born up to 1969 (when parental age was decreasing) and for sons born from 1970 (when parental age was increasing). [a]Continuous outcomes. Associations are mean differences (95% CI) from linear regression with robust standard errors clustered by paternal identity. [b]Binary outcomes. Associations are odds ratios (95% CI) from logistic regression with robust standard errors clustered by paternal identity. Adjustment set (e) was used; a cubic spline of offspring DOB, SEP in both parents, birth order and maternal age at son's birth. Data on birth weight, birth length and smoking status were only available in one period and were thus omitted as outcomes.

	**Sample size (fathers)**		**Primary association per five years of father's age at son's birth**	
**Outcome**	**DOB<=1969**	**DOB>=1970**	**DOB<=1969**	**DOB>=1970**
Height at 18 (cm)[a]	884,453 (676,783)	698,429 (555,682)	0.02 (0.00, 0.04)	-0.09 (-0.11, -0.06)
BMI at 18 (kg m^{-2})[a]	884,127 (676,614)	698,403 (555,665)	0.00 (0.00, 0.01)	0.03 (0.01, 0.04)
SBP at 18 (mmHg)[a]	884,385 (676,741)	622,964 (504,956)	0.15 (0.12, 0.18)	0.02 (-0.02, 0.06)
DBP at 18 (mmHg)[a]	884,320 (676,708)	622,820 (504,860)	0.02 (-0.01, 0.04)	0.10 (0.07, 0.13)
Intelligence at 18 (1-9)[a]	893,160 (682,106)	745,383 (586,213)	0.06 (0.06, 0.07)	0.00 (-0.01, 0.00)
Non-cognitive ability at 18 (1-9)[a]	812,510 (631,120)	263,069 (233,491)	0.00 (0.00, 0.01)	-0.05 (-0.05, -0.04)
Non-manual employment[b]	721,947 (575,289)	124,032 (120,733)	1.01 (1.01, 1.02)	0.97 (0.95, 1.00)
Full secondary education[b]	981,008 (728,108)	874,036 (665,917)	1.08 (1.07, 1.08)	1.00 (0.99, 1.00)
Left-handed[b]	861,342 (663,583)	438,755 (372,304)	1.00 (0.99, 1.01)	1.00 (0.98, 1.01)

Supplementary Table S10. Sibling comparison analyses of son outcomes and maternal age, conducted separately for sons born up to 1969 (when parental age was decreasing) and for sons born from 1970 (when parental age was increasing). [a]Continuous outcomes. Associations are mean differences (95% CI) from fixed-effects linear regression grouped by maternal identity. [b]Binary outcomes. Associations are odds ratios (95% CI) from conditional logistic regression grouped by maternal identity. Adjustment set (e) was used; paternal SEP, birth order, and paternal age at son's birth.

	**Sample size (mothers)**		**Sibling-comparison association per five years of mother's age at son's birth**	
**Outcome**	**DOB<=1969**	**DOB>=1970**	**DOB<=1969**	**DOB>=1970**
Height at 18 (cm)[a]	376,567 (171,967)	268,675 (126,614)	0.68 (0.60, 0.77)	0.72 (0.61, 0.83)
BMI at 18 (kg m^{-2})[a]	376,285 (171,843)	268,656 (126,605)	0.51 (0.47, 0.55)	0.74 (0.67, 0.80)
SBP at 18 (mmHg)[a]	376,516 (171,945)	223,379 (105,826)	0.68 (0.49, 0.87)	1.16 (0.88, 1.44)
DBP at 18 (mmHg)[a]	376,462 (171,923)	223,276 (105,777)	-2.68 (-2.84, -2.51)	2.16 (1.91, 2.41)
Intelligence at 18 (1-9)[a]	382,422 (174,491)	298,635 (140,020)	0.04 (0.01, 0.07)	0.07 (0.04, 0.10)
Non-cognitive ability at 18 (1-9)[a]	329,711 (151,299)	57,453 (28,118)	0.06 (0.03, 0.09)	-0.14 (-0.26, -0.02)
Full secondary education[b]	161,917 (70,474)	114,028 (50,795)	1.16 (1.11, 1.21)	1.89 (1.80, 1.98)
Left-handed[b]	65,598 (28,679)	18,720 (8,958)	0.91 (0.85, 0.98)	1.07 (0.89, 1.30)

Supplementary Table S11. Sibling comparison analyses of son outcomes and paternal age, conducted separately for sons born up to 1969 (when parental age was decreasing) and for sons born from 1970 (when parental age was increasing). [a]Continuous outcomes. Associations are mean differences (95% CI) from fixed-effects linear regression grouped by paternal identity. [b]Binary outcomes. Associations are odds ratios (95% CI) from conditional logistic regression grouped by paternal identity. Adjustment set (e) was used; maternal SEP, birth order, and maternal age at son's birth.

	**Sample size (fathers)**		**Sibling-comparison association per five years of father's age at son's birth**	
**Outcome**	**DOB<=1969**	**DOB>=1970**	**DOB<=1969**	**DOB>=1970**
Height at 18 (cm)[a]	380,681 (173,011)	269,302 (126,555)	0.51 (0.44, 0.58)	0.41 (0.29, 0.52)
BMI at 18 (kg m^{-2})[a]	380,403 (172,890)	269,286 (126,548)	0.36 (0.32, 0.39)	0.56 (0.49, 0.62)
SBP at 18 (mmHg)[a]	380,640 (172,996)	223,775 (105,767)	0.62 (0.46, 0.77)	1.12 (0.84, 1.40)
DBP at 18 (mmHg)[a]	380,587 (172,975)	223,687 (105,727)	-2.63 (-2.78, -2.49)	1.72 (1.47, 1.97)
Intelligence at 18 (1-9)[a]	386,589 (175,535)	298,896 (139,726)	-0.06 (-0.08, -0.03)	-0.02 (-0.06, 0.01)
Non-cognitive ability at 18 (1-9)[a]	333,970 (152,580)	57,864 (28,286)	0.15 (0.12, 0.17)	-0.03 (-0.15, 0.09)
Full secondary education[b]	167,098 (72,053)	115,547 (51,179)	1.04 (1.01, 1.08)	1.63 (1.56, 1.71)
Left-handed[b]	66,653 (28,983)	18,832 (8,994)	0.97 (0.92, 1.03)	0.96 (0.81, 1.15)

Supplementary Table S12. Analyses of son's outcomes and maternal age, with and without additional adjustment for maternal survival to the offspring's 16th birthday. [a]Continuous outcomes. Associations are mean differences (95% CI) from linear regression with robust standard errors clustered by maternal identity. [b]Binary outcomes. Associations are odds ratios (95% CI) from logistic regression with robust standard errors clustered by maternal identity. Adjustment set (e) was used; a cubic spline of offspring DOB, SEP in both parents, birth order and paternal age at son's birth.

		Primary association per five years of mother's age at son's birth	
**Outcome**	**N (mothers)**	**Without lifespan overlap**	**With lifespan overlap**
Height at 18 (cm)[a]	1,536,176 (1,134,355)	0.42 (0.41, 0.44)	0.43 (0.41, 0.44)
BMI at 18 (kg m-2)[a]	1,535,836 (1,134,199)	0.00 (-0.01, 0.01)	0.00 (-0.01, 0.01)
SBP at 18 (mmHg)[a]	1,460,648 (1,085,515)	0.39 (0.36, 0.42)	0.39 (0.37, 0.42)
DBP at 18 (mmHg)[a]	1,460,425 (1,085,427)	0.13 (0.11, 0.15)	0.13 (0.10, 0.15)
Intelligence at 18 (1-9)a	1,591,528 (1,168,798)	0.19 (0.19, 0.19)	0.19 (0.19, 0.20)
Non-cognitive ability at 18 (1-9)[a]	1,028,946 (802,624)	0.08 (0.07, 0.08)	0.08 (0.07, 0.08)
Non-manual employment[b]	808,862 (644,380)	1.21 (1.20, 1.22)	1.21 (1.20, 1.22)
Full secondary education[b]	1,805,413 (1,284,511)	1.32 (1.31, 1.33)	1.32 (1.32, 1.33)
Left-handed[b]	1,253,664 (942,979)	0.97 (0.96, 0.98)	0.97 (0.96, 0.98)

Supplementary Table S13. Analyses of son's outcomes and paternal age, with and without additional adjustment for paternal survival to the offspring's 16th birthday. [a]Continuous outcomes. Associations are mean differences (95% CI) from linear regression with robust standard errors clustered by paternal identity. [b]Binary outcomes. Associations are odds ratios (95% CI) from logistic regression with robust standard errors clustered by paternal identity. Adjustment set (e) was used; a cubic spline of offspring DOB, SEP in both parents, birth order and maternal age at son's birth.

		Primary association per five years of father's age at son's birth	
**Outcome**	**N (fathers)**	**Without lifespan overlap**	**With lifespan overlap**
Height at 18 (cm)[a]	1,536,160 (1,127,773)	-0.02 (-0.04, -0.01)	-0.02 (-0.03, -0.01)
BMI at 18 (kg m-2)[a]	1,535,820 (1,127,612)	0.01 (0.00, 0.02)	0.01 (0.00, 0.01)
SBP at 18 (mmHg)[a]	1,460,632 (1,079,692)	0.09 (0.07, 0.11)	0.09 (0.07, 0.11)
DBP at 18 (mmHg)[a]	1,460,409 (1,079,589)	0.04 (0.02, 0.06)	0.04 (0.02, 0.06)
Intelligence at 18 (1-9)a	1,591,510 (1,161,999)	0.03 (0.02, 0.03)	0.03 (0.03, 0.03)
Non-cognitive ability at 18 (1-9)[a]	1,028,928 (798,170)	-0.01 (-0.02, -0.01)	-0.01 (-0.02, -0.01)
Non-manual employment[b]	808,846 (641,571)	1.01 (1.00, 1.02)	1.01 (1.00, 1.02)
Full secondary education[b]	1,805,393 (1,276,470)	1.04 (1.04, 1.05)	1.05 (1.04, 1.05)
Left-handed[b]	1,253,649 (938,651)	1.00 (0.99, 1.00)	1.00 (0.99, 1.00)

Supplementary Table S14. Primary analyses of son outcomes and maternal age, with and without additional adjustment for offspring birth weight and birth length. [a]Continuous outcomes. Associations are mean differences (95% CI) from linear regression with robust standard errors clustered by maternal identity. [b]Binary outcomes. Associations are odds ratios (95% CI) from logistic regression with robust standard errors clustered by maternal identity. Adjustment set (e) was used; a cubic spline of offspring DOB, SEP in both parents, birth order and paternal age at son's birth.

		**Primary association per five years of mother's age at son's birth**	
**Outcome**	**N (mothers)**	**Without birth weight & length**	**With birth weight & length**
Height at 18 (cm)[a]	540,599 (442,964)	0.33 (0.30, 0.36)	0.35 (0.32, 0.38)
BMI at 18 (kg m-2)[a]	540,582 (442,952)	-0.02 (-0.04, 0.00)	-0.01 (-0.03, 0.00)
SBP at 18 (mmHg)[a]	467,278 (390,847)	0.37 (0.31, 0.43)	0.36 (0.30, 0.42)
DBP at 18 (mmHg)[a]	467,206 (390,795)	0.22 (0.17, 0.28)	0.22 (0.17, 0.27)
Intelligence at 18 (1-9)a	585,718 (473,117)	0.16 (0.15, 0.17)	0.16 (0.15, 0.17)
Non-cognitive ability at 18 (1-9)[a]	166,102 (153,716)	0.02 (0.00, 0.03)	0.02 (0.00, 0.03)
Full secondary education[b]	701,490 (548,117)	1.31 (1.29, 1.32)	1.31 (1.29, 1.32)
Left-handed[b]	283,375 (254,764)	0.98 (0.96, 1.00)	0.98 (0.96, 1.00)

Supplementary Table S15. Primary analyses of son outcomes and paternal age, with and without additional adjustment for offspring birth weight and birth length. [a]Continuous outcomes. Associations are mean differences (95% CI) from linear regression with robust standard errors clustered by maternal identity. [b]Binary outcomes. Associations are odds ratios (95% CI) from logistic regression with robust standard errors clustered by maternal identity. Adjustment set (e) was used; a cubic spline of offspring DOB, SEP in both parents, birth order and paternal age at son's birth.

		**Primary association per five years of father's age at son's birth**	
**Outcome**	**N (fathers)**	**Without birth weight & length**	**With birth weight & length**
Height at 18 (cm)[a]	540,599 (442,793)	-0.10 (-0.12, -0.07)	-0.08 (-0.11, -0.06)
BMI at 18 (kg m-2)[a]	540,582 (442,781)	0.03 (0.02, 0.04)	0.03 (0.02, 0.04)
SBP at 18 (mmHg)[a]	467,278 (390,749)	0.02 (-0.02, 0.07)	0.02 (-0.02, 0.07)
DBP at 18 (mmHg)[a]	467,206 (390,697)	0.12 (0.08, 0.16)	0.12 (0.08, 0.16)
Intelligence at 18 (1-9)a	585,718 (473,137)	-0.01 (-0.02, 0.00)	-0.01 (-0.02, 0.00)
Non-cognitive ability at 18 (1-9)[a]	166,102 (153,600)	-0.05 (-0.06, -0.04)	-0.05 (-0.06, -0.04)
Full secondary education[b]	701,490 (548,151)	0.97 (0.96, 0.98)	0.97 (0.96, 0.98)
Left-handed[b]	283,375 (254,544)	1.01 (0.99, 1.03)	1.01 (0.99, 1.03)

Supplementary Table S16. Sibling comparison analyses of son outcomes and maternal age, with and without additional adjustment for offspring birth weight and birth length. [a]Continuous outcomes. Associations are mean differences (95% CI) from linear regression with robust standard errors clustered by maternal identity. [b]Binary outcomes. Associations are odds ratios (95% CI) from logistic regression with robust standard errors clustered by maternal identity. Adjustment set (e) was used; a cubic spline of offspring DOB, SEP in both parents, birth order and paternal age at son's birth.

		Sibling-comparison association per five years of mother's age at son's birth	
**Outcome**	**N (mothers)**	**Without birth weight & length**	**With birth weight & length**
Height at 18 (cm)[a]	186,056 (88,421)	0.76 (0.61, 0.92)	0.78 (0.63, 0.93)
BMI at 18 (kg m-2)[a]	186,046 (88,416)	0.71 (0.62, 0.80)	0.71 (0.62, 0.80)
SBP at 18 (mmHg)[a]	146,383 (69,952)	1.14 (0.73, 1.55)	1.14 (0.73, 1.55)
DBP at 18 (mmHg)[a]	146,348 (69,937)	2.38 (2.03, 2.73)	2.38 (2.03, 2.73)
Intelligence at 18 (1-9)a	213,492 (100,891)	0.06 (0.02, 0.10)	0.06 (0.02, 0.10)
Non-cognitive ability at 18 (1-9)[a]	24,562 (12,176)	-0.41 (-0.69, -0.14)	-0.42 (-0.70, -0.15)
Full secondary education[b]	74,155 (33,407)	1.49 (1.40, 1.59)	1.49 (1.40, 1.59)
Left-handed[b]	7,829 (3,848)	1.25 (0.83, 1.89)	1.26 (0.83, 1.90)

Supplementary Table S17. Sibling comparison analyses of son outcomes and paternal age, with and without additional adjustment for offspring birth weight and birth length. [a]Continuous outcomes. Associations are mean differences (95% CI) from linear regression with robust standard errors clustered by maternal identity. [b]Binary outcomes. Associations are odds ratios (95% CI) from logistic regression with robust standard errors clustered by maternal identity. Adjustment set (e) was used; a cubic spline of offspring DOB, SEP in both parents, birth order and paternal age at son's birth.

		Sibling-comparison association per five years of father's age at son's birth	
**Outcome**	**N (fathers)**	**Without birth weight & length**	**With birth weight & length**
Height at 18 (cm)[a]	186,141 (88,335)	0.57 (0.41, 0.73)	0.60 (0.45, 0.75)
BMI at 18 (kg m-2)[a]	186,131 (88,330)	0.54 (0.44, 0.63)	0.53 (0.43, 0.62)
SBP at 18 (mmHg)[a]	146,429 (69,900)	1.23 (0.80, 1.65)	1.24 (0.81, 1.66)
DBP at 18 (mmHg)[a]	146,394 (69,885)	2.10 (1.73, 2.46)	2.10 (1.74, 2.46)
Intelligence at 18 (1-9)a	213,141 (100,560)	0.02 (-0.02, 0.07)	0.02 (-0.03, 0.07)
Non-cognitive ability at 18 (1-9)[a]	24,775 (12,273)	-0.21 (-0.48, 0.05)	-0.22 (-0.48, 0.05)
Full secondary education[b]	74,721 (33,559)	1.34 (1.27, 1.42)	1.34 (1.27, 1.42)
Left-handed[b]	7,915 (3,889)	1.20 (0.84, 1.69)	1.20 (0.85, 1.70)

Supplementary Table S18. Associations between son outcomes and maternal age from primary and sibling comparison analyses with families defined by both parents' identities. Primary analyses and the secular trends analyses used linear or logistic regression with robust standard errors clustered by both parents' identities. The sibling-comparison analysis used fixed-effects linear or conditional logistic regression grouped by both parents' identities. Adjustment set (e) (offspring DOB, maternal and paternal occupational and educational SEP, offspring birth order, and paternal age) was used for all analyses, except that offspring DOB was replaced by maternal DOB where indicated and that parent-level terms and offspring DOB were omitted in the sibling-comparison analysis. Restricted data, a necessity for the sibling comparison analysis, consisted of those sons who had a brother in the dataset. Offspring of both sexes were used for the analysis of sex. [a]Continuous outcomes. Associations are mean differences (95% CI). [b]Binary outcomes. Associations are odds ratios (95% CI).

	**Association per five years of mother's age at son's birth (or per five years of son's DOB for secular trend)**				
**Outcome**	**Primary analysis**	**Primary Analysis (mother's DOB)**	**Primary Analysis (mother's DOB, restricted data)**	**Sibling-comparison analysis (restricted data)**	**Secular trend (restricted data)**
Height at 18 (cm)[a]	0.42 (0.40, 0.44)	0.61 (0.59, 0.63)	0.69 (0.66, 0.72)	0.56 (0.52, 0.59)	0.29 (0.28, 0.30)
BMI at 18 (kg m^{-2})[a]	0.00 (-0.01, 0.01)	0.29 (0.28, 0.30)	0.28 (0.27, 0.30)	0.47 (0.45, 0.49)	0.26 (0.25, 0.26)
SBP at 18 (mmHg)[a]	0.39 (0.36, 0.42)	0.80 (0.77, 0.83)	0.72 (0.66, 0.77)	0.49 (0.41, 0.58)	0.19 (0.18, 0.21)
DBP at 18 (mmHg)[a]	0.13 (0.11, 0.15)	-0.44 (-0.47, -0.42)	-0.64 (-0.68, -0.59)	-0.97 (-1.05, -0.89)	-0.64 (-0.65, -0.63)
Intelligence at 18 (1-9)[a]	0.19 (0.18, 0.19)	0.09 (0.08, 0.09)	0.12 (0.11, 0.13)	0.03 (0.01, 0.04)	0.00 (-0.01, 0.00)
Non-cognitive ability at 18 (1-9)[a]	0.08 (0.07, 0.08)	0.09 (0.09, 0.10)	0.14 (0.13, 0.15)	0.02 (0.00, 0.03)	0.15 (0.14, 0.15)
Birth weight (hg)[a]	-0.14 (-0.16, -0.11)	0.00 (-0.02, 0.03)	0.10 (0.05, 0.16)	-0.05 (-0.12, 0.01)	0.06 (0.03, 0.09)
Birth length (cm)[a]	-0.02 (-0.03, -0.01)	-0.04 (-0.05, -0.03)	-0.02 (-0.04, 0.01)	-0.07 (-0.10, -0.04)	-0.06 (-0.08, -0.05)
Non-manual employment[b]	1.21 (1.21, 1.22)	0.63 (0.62, 0.63)	0.71 (0.71, 0.72)	0.61 (0.59, 0.64)	0.79 (0.79, 0.80)
Full secondary education[b]	1.31 (1.31, 1.32)	1.74 (1.73, 1.75)	1.23 (1.22, 1.23)	1.64 (1.61, 1.68)	1.07 (1.07, 1.08)
Smoker at 18[b]	0.86 (0.83, 0.88)	0.68 (0.48, 0.95)			
Left-handed[b]	0.97 (0.96, 0.98)	0.95 (0.94, 0.96)	1.03 (1.02, 1.04)	0.92 (0.89, 0.96)	1.02 (1.02, 1.03)

Supplementary Table S19. Associations between son outcomes and paternal age from primary and sibling comparison analyses with families defined by both parents' identities. Primary analyses and the secular trends analyses used linear or logistic regression with robust standard errors clustered by both parents' identities. The sibling-comparison analysis used fixed-effects linear or conditional logistic regression grouped by both parents' identities. Adjustment set (e) (offspring DOB, maternal and paternal occupational and educational SEP, offspring birth order, and paternal age) was used for all analyses, except that offspring DOB was replaced by maternal DOB where indicated and that parent-level terms and offspring DOB were omitted in the sibling-comparison analysis. Restricted data, a necessity for the sibling comparison analysis, consisted of those sons who had a brother in the dataset. Offspring of both sexes were used for the analysis of sex. [a]Continuous outcomes. Associations are mean differences (95% CI). [b]Binary outcomes. Associations are odds ratios (95% CI).

	**Association per five years of father's age at son's birth (or per five years of son's DOB for secular trend)**				
**Outcome**	**Primary analysis**	**Primary Analysis (father's DOB)**	**Primary Analysis (father's DOB, restricted data)**	**Sibling-comparison analysis (restricted data)**	**Secular trend (restricted data)**
Height at 18 (cm)[a]	-0.02 (-0.03, -0.01)	0.16 (0.14, 0.17)	0.17 (0.14, 0.20)	0.56 (0.52, 0.59)	0.29 (0.28, 0.30)
BMI at 18 (kg m^{-2})[a]	0.01 (0.00, 0.02)	0.30 (0.29, 0.31)	0.31 (0.29, 0.32)	0.47 (0.45, 0.49)	0.26 (0.25, 0.26)
SBP at 18 (mmHg)[a]	0.10 (0.07, 0.12)	0.50 (0.48, 0.53)	0.44 (0.39, 0.48)	0.49 (0.41, 0.58)	0.19 (0.18, 0.21)
DBP at 18 (mmHg)[a]	0.04 (0.02, 0.06)	-0.76 (-0.78, -0.74)	-0.89 (-0.93, -0.85)	-0.97 (-1.05, -0.89)	-0.64 (-0.65, -0.63)
Intelligence at 18 (1-9)[a]	0.03 (0.02, 0.03)	-0.07 (-0.08, -0.07)	-0.06 (-0.07, -0.05)	0.03 (0.01, 0.04)	0.00 (-0.01, 0.00)
Non-cognitive ability at 18 (1-9)[a]	-0.01 (-0.02, -0.01)	0.00 (0.00, 0.01)	0.03 (0.02, 0.04)	0.02 (0.00, 0.03)	0.15 (0.14, 0.15)
Birth weight (hg)[a]	-0.04 (-0.06, -0.03)	0.09 (0.07, 0.12)	0.14 (0.08, 0.19)	-0.05 (-0.12, 0.01)	0.06 (0.03, 0.09)
Birth length (cm)[a]	-0.02 (-0.03, -0.02)	-0.05 (-0.06, -0.03)	-0.04 (-0.06, -0.01)	-0.07 (-0.10, -0.04)	-0.06 (-0.08, -0.05)
Non-manual employment[b]	1.01 (1.00, 1.02)	0.54 (0.53, 0.54)	0.75 (0.75, 0.76)	0.61 (0.59, 0.64)	0.79 (0.79, 0.80)
Full secondary education[b]	1.04 (1.04, 1.05)	1.35 (1.35, 1.36)	1.10 (1.10, 1.11)	1.64 (1.61, 1.68)	1.07 (1.07, 1.08)
Smoker at 18[b]	0.91 (0.89, 0.94)	0.74 (0.53, 1.04)			
Left-handed[b]	1.00 (0.99, 1.00)	0.98 (0.97, 0.99)	1.00 (0.99, 1.01)	0.92 (0.89, 0.96)	1.02 (1.02, 1.03)

Supplementary Table S20. Associations between son outcomes and maternal age from primary and sibling comparison analyses, including the potentially biased subset of sons who died between 1961 and 30th June 1991 (N=56,901). [a]Continuous outcomes. Associations are mean differences (95% CI). [b]Binary outcomes. Associations are odds ratios (95% CI). Primary analyses and the secular trends analyses used linear or logistic regression with robust standard errors clustered by maternal identity. The sibling-comparison analysis used fixed-effects linear or conditional logistic regression grouped by maternal identity. Adjustment set (e) (offspring DOB, maternal and paternal occupational and educational SEP, offspring birth order, and paternal age) was used for all analyses, except that offspring DOB was replaced by maternal DOB where indicated and that mother-level terms and offspring DOB were omitted in the sibling-comparison analysis. Restricted data, a necessity for the sibling comparison analysis, consisted of those sons who had a brother in the dataset.

	**Association per five years of mother's age at son's birth (or per five years of son's DOB for secular trend)**				
**Outcome**	**Primary analysis**	**Primary Analysis (mother's DOB)**	**Primary Analysis (mother's DOB, restricted data)**	**Sibling-comparison analysis (restricted data)**	**Secular trend (restricted data)**
Height at 18 (cm)[a]	0.42 (0.40, 0.44)	0.61 (0.59, 0.63)	0.69 (0.66, 0.71)	0.69 (0.65, 0.74)	0.25 (0.24, 0.26)
BMI at 18 (kg m^{-2})[a]	0.00 (-0.01, 0.01)	0.29 (0.28, 0.30)	0.30 (0.29, 0.31)	0.59 (0.56, 0.62)	0.29 (0.28, 0.29)
SBP at 18 (mmHg)[a]	0.39 (0.36, 0.42)	0.81 (0.78, 0.84)	0.74 (0.69, 0.79)	0.70 (0.59, 0.82)	0.24 (0.22, 0.25)
DBP at 18 (mmHg)[a]	0.13 (0.11, 0.15)	-0.45 (-0.47, -0.42)	-0.53 (-0.57, -0.49)	-0.20 (-0.30, -0.10)	-0.69 (-0.70, -0.67)
Intelligence at 18 (1-9)[a]	0.19 (0.18, 0.19)	0.09 (0.08, 0.09)	0.12 (0.12, 0.13)	0.07 (0.05, 0.08)	-0.05 (-0.06, -0.05)
Non-cognitive ability at 18 (1-9)[a]	0.08 (0.07, 0.08)	0.09 (0.09, 0.10)	0.15 (0.14, 0.15)	0.08 (0.05, 0.10)	0.09 (0.08, 0.09)
Birth weight (hg)[a]	-0.14 (-0.16, -0.11)	0.01 (-0.01, 0.04)	0.14 (0.09, 0.19)	-0.12 (-0.22, -0.03)	0.19 (0.15, 0.22)
Birth length (cm)[a]	-0.02 (-0.03, -0.01)	-0.03 (-0.04, -0.02)	0.01 (-0.01, 0.03)	-0.06 (-0.10, -0.02)	0.00 (-0.02, 0.01)
Non-manual employment[b]	1.21 (1.21, 1.22)	0.63 (0.62, 0.63)	0.71 (0.70, 0.72)	0.63 (0.60, 0.66)	0.79 (0.78, 0.79)
Full secondary education[b]	1.32 (1.31, 1.32)	1.74 (1.73, 1.75)	1.37 (1.35, 1.38)	1.86 (1.82, 1.91)	1.10 (1.10, 1.10)
Smoker at 18[b]	0.85 (0.83, 0.88)	0.67 (0.48, 0.94)			
Left-handed[b]	0.97 (0.96, 0.98)	0.95 (0.94, 0.96)	1.03 (1.02, 1.04)	0.90 (0.86, 0.94)	1.02 (1.01, 1.02)

Supplementary Table S21. Associations between son outcomes and paternal age from primary and sibling comparison analyses, including the potentially biased subset of sons who died between 1961 and 30th June 1991 (N=56,901). [a]Continuous outcomes. Associations are mean differences (95% CI). [b]Binary outcomes. Associations are odds ratios (95% CI). Primary analyses and the secular trends analyses used linear or logistic regression with robust standard errors clustered by paternal identity. The sibling-comparison analysis used fixed-effects linear or conditional logistic regression grouped by paternal identity. Adjustment set (e) (offspring DOB, maternal and paternal occupational and educational SEP, offspring birth order, and maternal age) was used for all analyses, except that offspring DOB was replaced by paternal DOB where indicated and that father-level terms and offspring DOB were omitted in the sibling-comparison analysis. Restricted data, a necessity for the sibling comparison analysis, consisted of those sons who had a brother in the dataset.

	**Association per five years of father's age at son's birth (or per five years of son's DOB for secular trend)**				
**Outcome**	**Primary analysis**	**Primary Analysis (father's DOB)**	**Primary Analysis (father's DOB, restricted data)**	**Sibling-comparison analysis (restricted data)**	**Secular trend (restricted data)**
Height at 18 (cm)[a]	-0.02 (-0.03, 0.00)	0.16 (0.14, 0.18)	0.17 (0.14, 0.19)	0.39 (0.35, 0.43)	0.16 (0.15, 0.17)
BMI at 18 (kg m^{-2})[a]	0.01 (0.00, 0.02)	0.30 (0.29, 0.31)	0.32 (0.31, 0.33)	0.40 (0.38, 0.42)	0.29 (0.28, 0.29)
SBP at 18 (mmHg)[a]	0.09 (0.07, 0.12)	0.50 (0.48, 0.53)	0.43 (0.39, 0.47)	0.63 (0.54, 0.72)	0.27 (0.25, 0.29)
DBP at 18 (mmHg)[a]	0.04 (0.02, 0.06)	-0.77 (-0.79, -0.74)	-0.76 (-0.79, -0.72)	-0.43 (-0.51, -0.35)	-0.78 (-0.79, -0.76)
Intelligence at 18 (1-9)[a]	0.03 (0.02, 0.03)	-0.07 (-0.08, -0.07)	-0.08 (-0.08, -0.07)	-0.07 (-0.09, -0.06)	-0.12 (-0.12, -0.12)
Non-cognitive ability at 18 (1-9)[a]	-0.01 (-0.02, -0.01)	0.01 (0.00, 0.01)	0.03 (0.02, 0.04)	0.12 (0.10, 0.14)	0.06 (0.05, 0.06)
Birth weight (hg)[a]	-0.05 (-0.07, -0.03)	0.10 (0.08, 0.13)	0.16 (0.11, 0.21)	0.20 (0.10, 0.30)	0.15 (0.11, 0.19)
Birth length (cm)[a]	-0.03 (-0.03, -0.02)	-0.04 (-0.05, -0.03)	-0.02 (-0.05, 0.00)	-0.01 (-0.06, 0.03)	-0.03 (-0.04, -0.01)
Non-manual employment[b]	1.01 (1.00, 1.02)	0.54 (0.53, 0.54)	0.70 (0.69, 0.71)	0.59 (0.57, 0.62)	0.76 (0.76, 0.77)
Full secondary education[b]	1.04 (1.04, 1.05)	1.36 (1.35, 1.36)	1.26 (1.25, 1.27)	1.44 (1.42, 1.47)	1.10 (1.10, 1.10)
Smoker at 18[b]	0.91 (0.89, 0.94)	0.73 (0.52, 1.03)			
Left-handed[b]	1.00 (0.99, 1.00)	0.98 (0.97, 0.99)	0.97 (0.96, 0.98)	0.94 (0.90, 0.97)	1.01 (1.01, 1.01)

Supplementary Figure S1. Unadjusted associations of paternal (filled circles) and maternal (open circles) age with factors among the parents of sons.

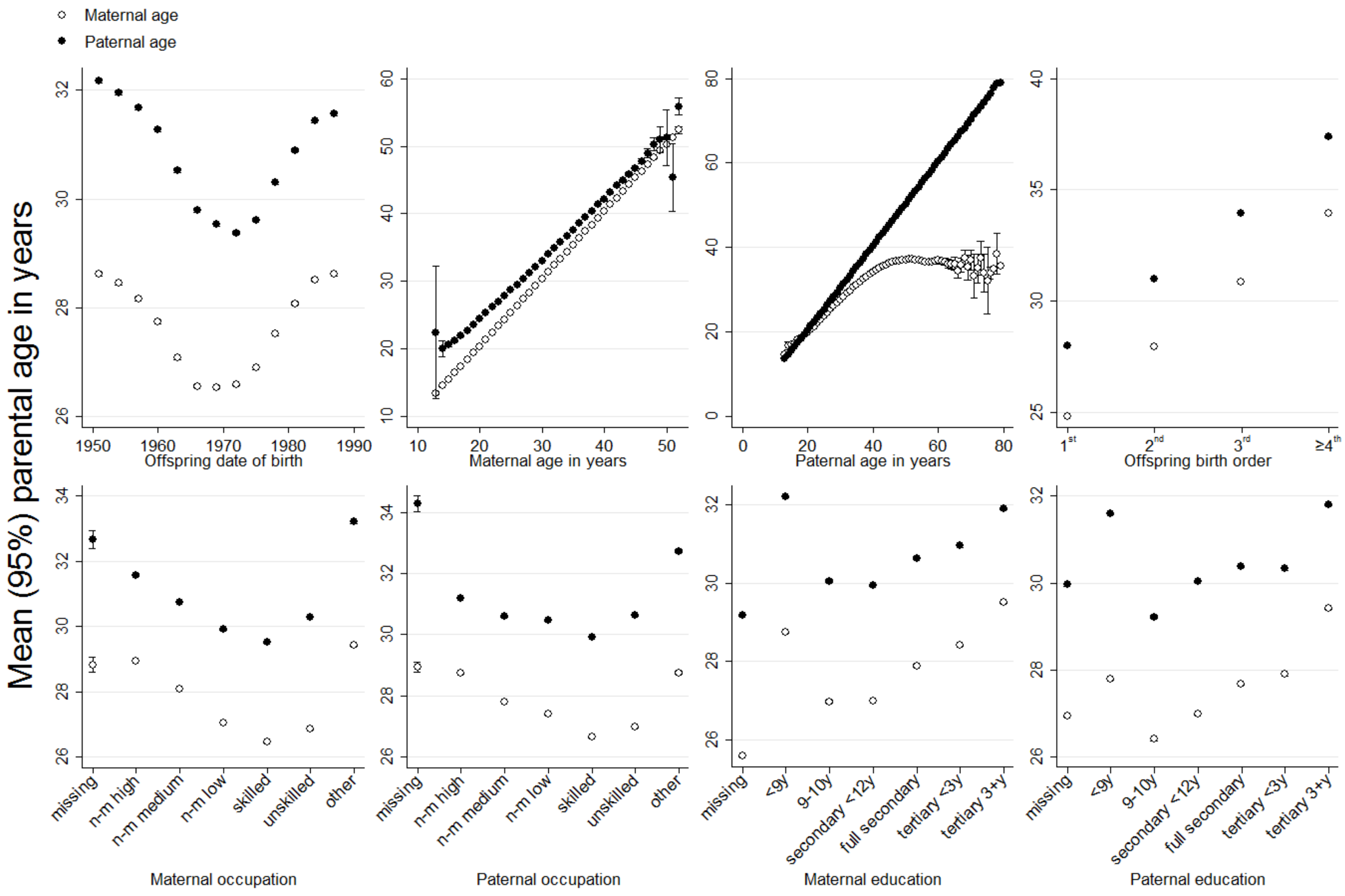

Supplementary Figure S2. Trends in parental age and outcome variables over the course of the study. 95% confidence intervals are shown where they exceed 10% of the plotted range. Trends for sons (black circles), daughters (white circles) or all offspring (grey circles) are plotted for three-year intervals of offspring date of birth, except for smoking, which is plotted for each year in which it was recorded. Trends for parental age were indistinguishable for sons and daughters (data not shown), so were plotted for all offspring.

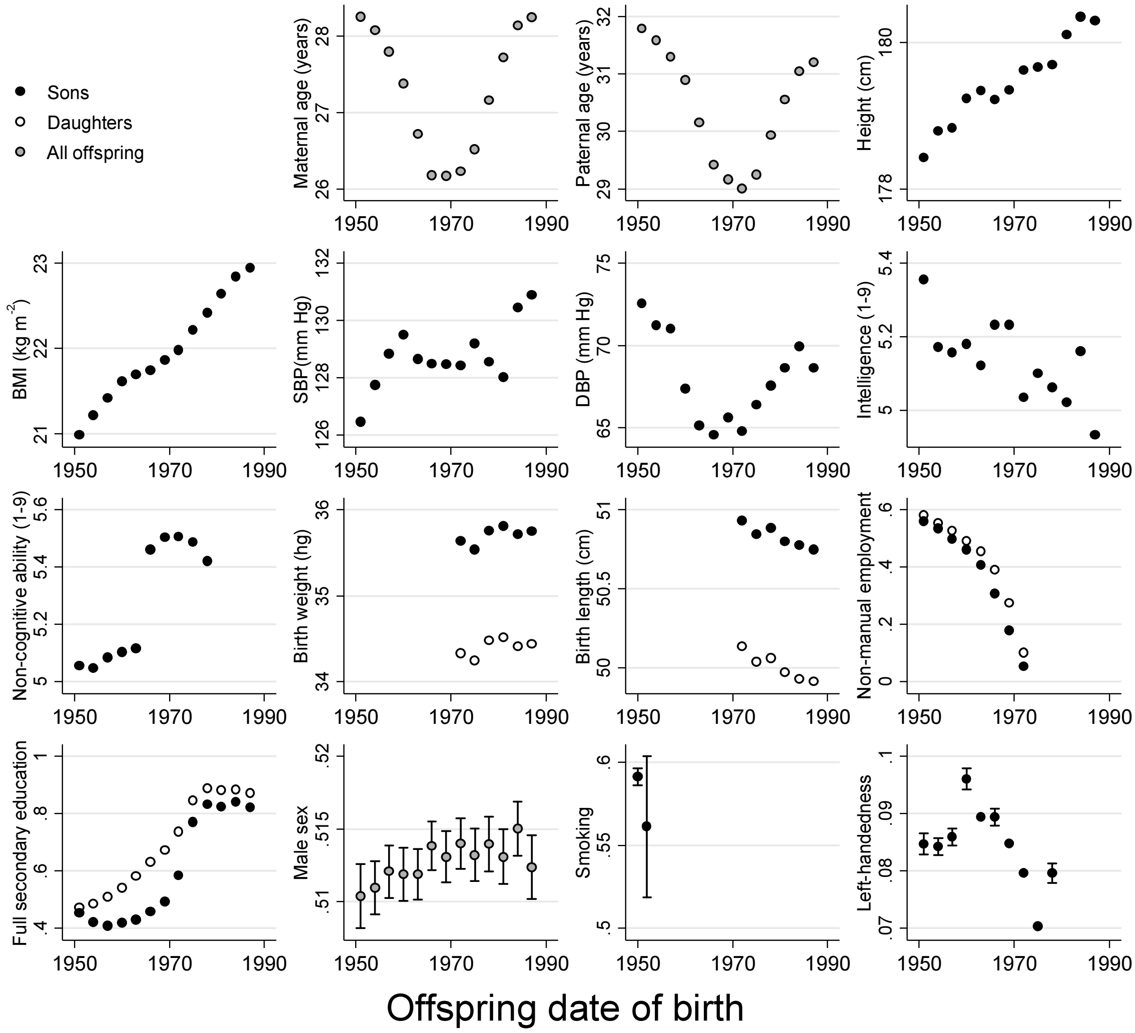

Supplementary Methods. Additional details of data preparation

Data Preparation – Conscription examinations

At each conscription medical examination, height and weight were recorded, and used to calculate BMI. Systolic (SBP) and diastolic (DBP) blood pressures were measured after 5-10 minutes' rest in a supine position. If SBP was less than or equal to 145 mmHg and DBP between 50 and 85 mmHg, a single measurement was made. Otherwise, a second measurement was made and this value was recorded and used for analysis[1]. Intelligence was recorded on a scale from 1 to 9 derived by combining the results from tests of synonyms, logic, spatial ability and technical knowledge[2,3]. Psychological aptitude for military service was scored on a scale of 1 to 9 following completion of a questionnaire and a 25-minute semi-structured interview by a psychologist[3]. We refer to this as non-cognitive ability because the characteristics which contribute to a high score include "willingness to assume responsibility; independence; outgoing character; persistence; emotional stability, and power of initiative"[3]. Handedness was recorded from September 1969 to March 1997 as the trigger hand on a weapon. From January 1984, only left-handedness was recorded, and we assumed those who underwent a conscription examination but lacked handedness data to be right-handed. With this assumption, sample sizes and rates of left-handedness were not noticeably different from those during the period when right-handedness was positively recorded. Monthly rates of left-handedness suggested that October-December 1983 was a transition period and handedness data from these months were excluded from analysis. Smoking habits were only recorded in 1969-1970, at 56% of the examinations in that period. 99 men with smoking data who were apparently examined in 1971-1976 had dates of birth indicating that they were probably examined in 1969-1970. They were not excluded because conscription year was not used in the analysis, and there was no evidence that their other data were incorrect. A binary outcome variable indicating current smoking was created.

Data Preparation – Swedish population and housing census.

The maximum attained educational level was recorded in 1970 and 1990 for parents and in 1970 and annually from 1990 to 2012 for offspring. We took the highest level attained and classified it into seven levels: <9 years; 9-10 years; incomplete secondary education; full secondary education; <3 years tertiary education; >=3 years tertiary education; and missing (1.0% of offspring, 18.5% of mothers and 15.8% of fathers). Occupational status was recorded in 1960, 1970, 1980 and 1990 with only the latter being available for offspring. We took the highest recorded status and classified it into seven levels; high non-manual; intermediate non-manual; low non-manual; skilled manual; unskilled manual; other; and missing. Occupational SEP was set to missing for offspring born after 1972 because many of them would still have been in education in 1990. It was also set to missing for parents born before 1895 because they would mostly have retired by 1960. Occupational status was missing in 40.5% of offspring, 0.2% of mothers and 0.5% of fathers. Among offspring born before 1973, 3.2% had missing occupational status data. Others (14.2% of offspring, 13.0% of mothers and 7.3% of fathers) included housewives, part-time workers, farmers and those students or pensioners not set to missing due to their age.

References

1 Sundström, J., Neovius, M., Tynelius, P. & Rasmussen, F. Association of blood pressure in late adolescence with subsequent mortality: cohort study of Swedish male conscripts. *Br. Med. J.* **342**, doi:d64310.1136/bmj.d643 (2011).
2 David, A. S., Malmberg, A., Brandt, L., Allebeck, P. & Lewis, G. IQ and risk for schizophrenia: a population-based cohort study. *Psychol. Med.* **27**, 1311-1323, doi:10.1017/s0033291797005680 (1997).
3 Lindqvist, E. & Vestman, R. The Labor Market Returns to Cognitive and Noncognitive Ability: Evidence from the Swedish Enlistment. *American Economic Journal-Applied Economics* **3**, 101-128, doi:10.1257/app.3.1.101 (2011).

Supplementary Note. Secular trends in sibling-comparison analyses of parental age. A simulation study of methodology.

Introduction

Sibling-comparison studies using fixed effects regression, conditional logistic regression, or stratified Cox regression have the great attraction of adjusting automatically for all measured and unmeasured family-level confounders. Such a property is particularly useful when the exposure of interest is parental age at the time of the offspring's birth. Because the exposure is determined before the birth of the offspring, there are few offspring-level variables which could conceivably influence both exposure and outcome, and most confounding is likely to take place at a family level. However, one severe drawback when applying sibling-comparison models to parental age is that offspring date of birth (DOB) is perfectly correlated with parental age within families. Adjustment for within-family secular trends in the outcome is therefore impossible, but the observed associations between parental age and the outcome may wholly or partly due to such trends[1]. Previous studies[2-4] have attempted to avoid this limitation by adjusting instead for offspring year of birth. Here, we use a simple simulation to show that this approach does not solve the problem and is vulnerable to additional confounding if the outcome varies according to season of birth. To conclude more positively, we consider (i) the useful interpretation that may be made of a sibling-comparison analysis without adjustment for offspring DOB and (ii) how to determine whether a conventional analysis, in which full adjustment for secular trends is possible, can be used in place of the sibling comparison analysis.

Methods

Data were simulated according to the DAG in Fig 1. Family-level data were simulated first, for 5,000,000 families. For simplicity, only one parent was simulated. The number of offspring was drawn from a Poisson distribution with a mean of 2.5 and the parent's date of birth, DOB_j, from a uniform distribution between 1900 and 1950. A family-level unmeasured confounder U_j was drawn from a standard normal distribution. The parent's age at the birth of their first offspring, PA_j, was a function of DOB_j and U_j, giving values of PA_j ranging between 8.8 and 33.0 with a mean of 21.2:

$$PA_j = -75 + 2*U_j + 0.05*DOB_j + Normal(0,1)$$

These family-level data were then replicated once for each offspring (some families, with no offspring, were thus removed entirely). For the first offspring in each family, parental age, PA_i, was equal to PA_j. Thereafter, birth intervals were drawn as 0.75+exp(0.5+0.75*Normal(0,1)), truncated to 30. This gave a right-skewed distribution with a theoretical minimum of 0.75, a median of 2.40 and an interquartile range of [1.74, 3.48]. Each offspring's date of birth, DOB_i, was then calculated as the sum of DOB_j and PA_i. DOB_i was rounded down to the nearest integer to give the offspring's year of birth, YOB_i. Versions of DOB_i rounded conventionally to 0, 1, 2 and 3 decimal places were also calculated.

Season of birth (SOB_i) was generated as a sine wave of amplitude 1 and wavelength 1 originating at the start of the year (Fig 2). Finally, a hypothetical outcome, Y_i, was calculated as a function of U_j, PA_i, DOB_i and SOB_i:

$$Y_i = \beta_{YiUj} *U_j + \beta_{YiPAi} *PA_i + 0.05*DOB_i + \beta_{YiSOBi}*SOB_i + Normal(0,1)$$

The coefficient for PA_i, β_{YiPAi}, was set to 0.1 and the coefficient for U_j, β_{YiUj} was set to 0.5. The coefficient for SOB_i, β_{YiSOBi} was set to zero in the first simulation. In the second and third, it was set to 0.02. Additionally, in the third simulation β_{YiUj} was set to 0 to remove the family-level confounding.

The association between Y_i and PA_i in the resulting data was analysed by linear regression with robust standard errors clustered by parental identity and by fixed effects regression grouped by parental identity (i.e. sibling-comparison analysis). Various adjustment sets including DOB_i, DOB_j and/or U_j were included (Table 1). Linear regression restricted to non-firstborn children, adjusted for DOB_j has been proposed[5] as an alternative means of separating family-level confounding from causal parental age effects, and was also used on these data. Since parental age is often analysed as categories, sibling-comparison analyses of the data from the first simulation were also conducted with indicator variables representing PA_i values in whole years with ≤19 and >=30 grouped and 24 as the reference category. The coefficient for 25 year olds was used for illustration, since it is expected to be approximately equal to the equivalent per-year continuous coefficient (since there is a one year interval from the reference group, and the within-group distribution of ages is relatively uniform in these groups). Simulations and analyses were run using Stata 13.1 (64-bit edition).

Results

The first three simulations each produced 12,500,491 offspring in 4,589,268 families. Of these, 7,911,223 offspring in 3,563,925 families were not the eldest offspring in the family and were thus included in the analyses adjusted for parental age at the birth of their first offspring. Although all offspring were used in the fixed-effects regression analysis, only those with siblings (11,475,148 offspring from 3,563,925 families) influenced the estimates of $\hat{\beta}_{YiPAi}$.

Results in the absence of a seasonal effect on Y_i are shown in Table 1A. The direct causal effect of PA_i on Y_i was recovered in a linear regression adjusted for both DOB_i and U_j (model A4), but estimates were biased if either or both of these variables was omitted (models A1-A3). Similarly, the linear regression model adjusted for PA_j recaptured the direct causal effect of PA_i on Y_i only when adjustment for DOB_i and U_j were included (models A5-A8). The coefficient in the unadjusted sibling-comparison analysis (model A9) was 0.150, equal to the sum of the direct simulated effect of PA_i on Y_i, and secular trend in Y_i. Attempted adjustment of this model for DOB_i (model A10) gave an identical estimate because Stata correctly identified that PA_i and DOB_i were perfectly co-linear within families and did not estimate a coefficient for the latter. When adjustment was for YOB_i (model A11) instead of DOB_i, the adjustment term was estimated, but its coefficient was imprecise and took an average value of zero over large numbers of simulations (results not shown). The supposedly adjusted estimate of $\hat{\beta}_{YiPAi}$ from model A11 took the same value as it did without the adjustment for YOB_i, but with wider confidence intervals. Modeling YOB nonlinearly as indicator variables (model A12) made no difference to this estimate. The same was true when DOB_i was conventionally rounded to the nearest year (model A13). Increasing the precision of this rounded version of DOB_i reduced the precision of the estimated $\hat{\beta}_{YiPAi}$ (models A14-A16), but when large numbers of simulations were performed, it remained centred on 0.150 (results not shown). When PA_i was analysed alone in categories, the coefficient for 25 year old mothers (relative to 24 year olds) was 0.151 (0.147, 0.154), once

again equal to the sum of the direct simulated effect of PA_i on Y_i, and secular trend in Y_i. When the analysis was adjusted for DOB_i, the coefficient for 25 year olds (and all categories of PA_i) was indistinguishable from the null but the coefficient for DOB_i was equal to the sum of the direct effect of PAi and the secular trend at 0.150 (0.149, 0.150). When adjusted for linear or categorical YOB_i, the coefficient was greater than the null, but considerably less than the simulated causal parameter β_{YiPAi} (0.006 (0.002, 0.009) and 0.007 (0.003, 0.010) for linear and categorical YOB_i, respectively), with the adjustment for secular trends again accounting for both the simulated secular trends and the direct causal effect of PA_i.

When a seasonal pattern in Y_i (Fig 2) was superimposed on the unchanged long-term trend (Table 1B), the unadjusted fixed effects model (model B9) gave the same result as it did without the seasonality. Adjustment for YOB_i, however (model B11), reduced the estimate by 0.039. This is approximately equal to the within-year secular trend (-0.038) in Y_i if the seasonal sine wave is represented linearly. When DOB_i was conventionally rounded to the nearest integer (model B13), the within-year linear secular trend became +0.038 and estimates of $\widehat{\beta}_{YiPAi}$ were increased by approximately this amount.

When there was seasonality, but no confounding by U_j (Table 1C), adjustment for DOB_i alone was sufficient to recover the direct causal effect of PA_i on Y_i in a linear regression (model C2). More notably, an unadjusted fixed-effects regression (model C9) gave the same estimate as a linear regression adjusted for the *parent's* DOB, DOB_j (model C17). This was not the case in the presence of confounding by U_j (models A9 and A17).

Discussion

Sibling-comparison analyses have been proposed to isolate the direct causal effect of parental age on offspring outcomes[2-4]. In the presence of secular trends in the outcome, however, we have shown that unadjusted estimates made by this method (model A9) represent the sum of the direct effect of parental age on the outcome, and an indirect causal effect mediated by offspring DOB (Fig 2). Because parental age and offspring DOB are perfectly co-linear within families, the contribution of the mediated pathway to the estimated coefficient $\widehat{\beta}_{YiPAi}$ is exactly equal to the secular trend in the outcome. Moreover, this perfect co-linearity means that offspring DOB (i.e. secular trends in the outcome) cannot be adjusted for. Offspring year of birth is merely a low-precision version of offspring DOB and the chosen level of imprecision (rounded down to the nearest year) is arbitrary. In the absence of seasonality, we show that adjustment for offspring DOB, however it is rounded or used as indicator variables, does not asymptotically change the estimated coefficient $\widehat{\beta}_{YiPAi}$, but only reduces its precision. When there is seasonal variation in the outcome, adjusting for a rounded form of offspring DOB biases the estimated coefficient $\widehat{\beta}_{YiPAi}$ by an amount equal to the linear within-year secular trend in the outcome. This trend depends critically on the arbitrary choice of when one year ends and the next begins, as can be seen by comparing models B11 and B13. When parental age was represented as categories and adjusted for year of birth, instead of the adjustment term being null and the parental age term accounting for both simulated effects, the opposite happened. This is probably because year of birth better fits the combined simulated effects than the categorised parental age. Whichever term is estimated, it appears that they cannot be distinguished in a sibling-comparison analysis.

Linear regression models adjusted for the parent's age at the birth of their first offspring and restricted to the analysis of subsequent offspring have been proposed as an alternative method to isolate the direct causal effect of parental age on offspring

outcomes[5]. As for sibling-comparison analyses, we show that in the presence of secular trends in the outcome, estimates of the coefficient $\hat{\beta}_{YiPAi}$ comprise a direct causal effect and an indirect effect mediated by offspring DOB (model A5). Once again, adjustment for offspring DOB (model A6) does not solve the problem. We interpret the residual bias in model A6 as resulting from a doubly-induced pathway (Fig 3d) in which adjustment for offspring DOB (DOB_i) induces association between its two causes parental age (PA_i) and parental DOB (DOB_j). Adjustment for parental age at the birth of their first offspring (PA_j) induces association between its causes parental DOB and the family-level confounding (U_j), opening a pathway from parental age to the offspring outcome via parental DOB and family-level confounders. Note also that in the present simulation, model A5 gave an identical estimate to model A9, equal to the combined direct and indirect causal effect of parental age on the offspring outcome. This was because (i) no intrinsic differences between oldest and subsequent offspring were simulated and (ii) all the simulated family-level confounding was mediated by parental age at the birth of their first offspring. This equivalence would not necessarily apply in real data.

In this simulation, the direct causal effect of parental age on the offspring outcome could only be recovered if the unmeasured confounding was absent (model C2) or measured (model A4). The first situation represents an untested assumption and the second is logically impossible. What then is to be done when we suspect both unmeasured family-level confounding and secular trends in the outcome, in an analysis of parental age? Sibling-comparison analysis accounts for all confounding at the family level, including family-level confounding by secular trends. What remains, in addition to the direct causal effect of parental age on the outcome, is an indirect causal effect of parental age on the outcome, mediated by offspring DOB (Fig 1). Since we cannot remove this indirect pathway in a fixed effects analysis, it is worth asking if we really want to. In the environment in which the data were collected, it is part of the causal effect of parental age on the offspring outcome. If a person decides to delay parenthood, their offspring's outcomes will differ in part because they have been born at a later time in a changing world[4]. The disadvantage of interpreting the full (i.e. direct and mediated) causal association is that it is contingent on the background trend. If the secular trend changed, the measured association would no longer apply. We may also be interested in isolating specific biological mechanisms, which would require the exclusion of such a pathway. The present results show that exclusion of the mediated pathway requires a return to linear regression (models A2 & C2), bringing with it the risk of family-level confounding. This risk may be reduced by excluding each parent's first offspring and adjusting for PA_j (model A6), but at the cost of potential induced confounding, and the risks of using a potentially unrepresentative subset of the data. We propose another analysis which may allow us to assess the extent of unmeasured family-level confounding, namely linear regression with adjustment for the *parent's* DOB (models A17 & C17). This leaves open the same direct and indirect causal pathways as the unadjusted sibling comparison analysis (models A9 & C9), but additionally allows family-level confounding except for family-level confounding by secular trends. Hence, if this analysis gives similar estimates to the unadjusted sibling comparison analysis (as for models C9 & C17), we may tentatively conclude that family-level confounding (other than by secular trends) is minimal. We could thus use a linear regression model adjusted for offspring DOB to estimate the direct causal effect of parental age, and an unadjusted fixed effects model to estimate its total causal effect. Such an approach would not be without assumptions. In particular, such an analysis could still be confounded by family-level secular trends, if their

effect on the outcome is not mediated by offspring DOB. Note that adjustment for both parental and offspring DOB is impossible because they completely define parental age.

In conclusion, sibling-comparison analyses of parental age and offspring outcomes have sometimes been adjusted for offspring year of birth, which is a reduced-precision form of offspring date of birth. This does not block the pathway from parental age to offspring outcomes that is mediated by offspring DOB. Estimates are made with reduced precision and may incorporate seasonal patterns in the outcome. If parental age is converted to categories, such an analysis may give a falsely null result, with the direct effect of parental age incorporated into the adjustment for secular trends. We point out that (i) a sibling-comparison association with parental age which is not adjusted for offspring DOB may still be causal, but includes a causal pathway mediated by offspring DOB, and (ii) a linear regression analysis adjusted for the *parent's* DOB will match the unadjusted sibling comparison analysis if there is no unmeasured family-level confounding. If this is the case a linear regression analysis may be used, adjusted for offspring DOB, to estimate the direct causal effect of parental age on offspring outcomes.

References

1 Holt, G. B. Fixed-effects models and diagnosing psychiatric disorders. *Jama Psychiatry* **71**, 1077-1078 (2014).

2 D'Onofrio, B. M. *et al.* Paternal age at childbearing and offspring psychiatric and academic morbidity. *JAMA psychiatry* **71**, 432-438, doi:10.1001/jamapsychiatry.2013.4525 (2014).

3 Myrskylae, M., Silventoinen, K., Tynelius, P. & Rasmussen, F. Is later better or worse? Association of advanced parental age with offspring cognitive ability among half a million young swedish men. *Am. J. Epidemiol.* **177**, 649-655, doi:10.1093/aje/kws237 (2013).

4 Barclay, K. & Myrskylae, M. Advanced maternal age and offspring outcomes: Reproductive aging and counterbalancing period trends. *Population and development review* **42**, 69-94 (2016).

5 Petersen, L., Mortensen, P. B. & Pedersen, C. B. Paternal age at birth of first child and risk of schizophrenia. *Am. J. Psychiatry* **168**, 82-88, doi:10.1176/appi.ajp.2010.10020252 (2011).

Figure 1. A DAG representing simulated data for an offspring outcome (Y_i); date of birth in the parent (DOB_j) and offspring (DOB_i); parental age at the birth of their first offspring (PA_j) and the offspring in question (PA_i); and an unmeasured family-level confounder (U_j). DOB_i is entirely determined as the sum of DOB_j and PA_i. In the second model, the effect of DOB_i on Y_i was nonlinear, with a seasonal effect varying the linear long-term effect.

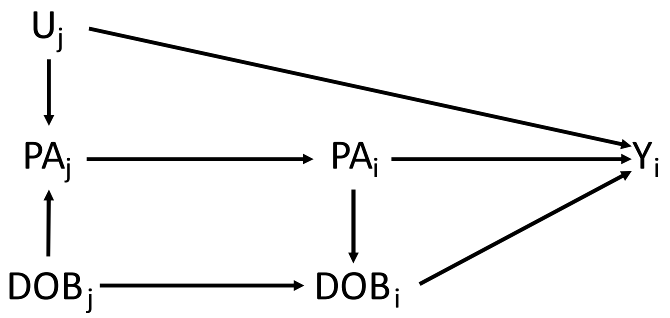

Figure 2. Temporal trends in offspring outcome Y_i. The total time trend (solid; left-hand axis) is the sum of a long-term trend (dashed; left-hand axis) and a seasonal pattern (dotted, right-hand axis).

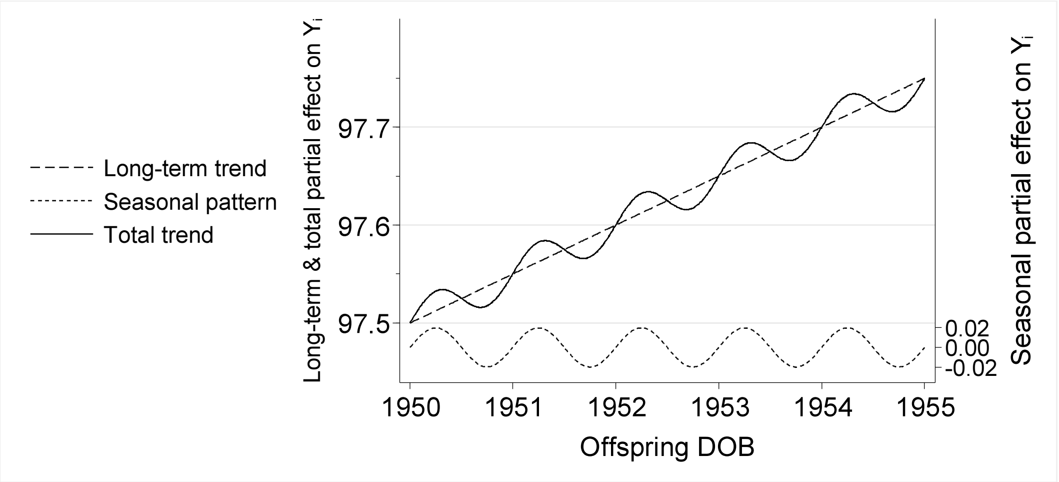

Figure 3. Copies of the DAG in Figure 1, with pathways from parental age to offspring outcome highlighted. Induced pathways are shown dotted. (1) Direct effect of parental age (PA_i) on outcome (Y_i). (2) Confounded pathway from PA_i to Y_i via family-level parental age (PA_j) and confounder (U_j). (3) Effect of PA_i on Y_i mediated by offspring date of birth (DOB_i). (4) Doubly-induced pathway from PA_i to Y_i via parental date of birth (DOB_j) and family-level confounder (U_j).

a) Linear regression, adjusted for offspring DOB (model 2)

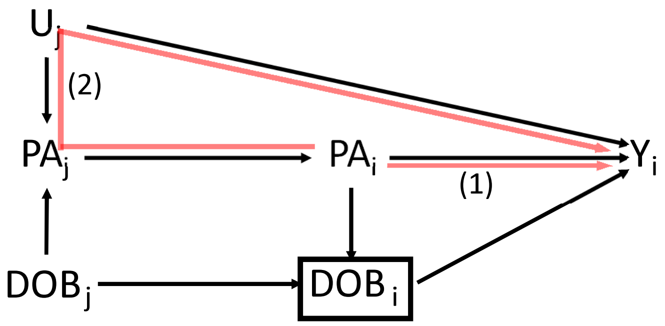

b) Linear regression, adjusted for parental DOB (model 17)

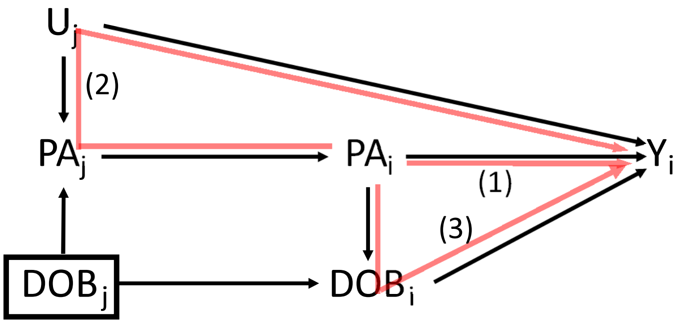

c) Unadjusted sibling-comparison analysis (model 9)

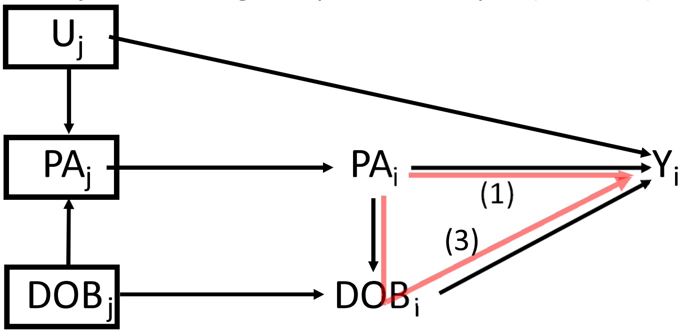

d) Linear regression, adjusted for parental age at the birth of their first offspring and for offspring DOB (model 6)

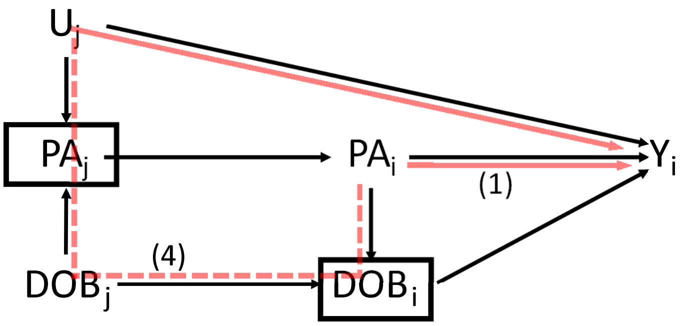

Table 1. Estimates of the association between offspring outcome Y_i and parental age PA_i. The direct causal association was simulated to be 0.100. The linear secular trend was 0.050. Seasonal variation in Y_i, where indicated, comprised a sine wave of wavelength 1.0 and amplitude 0.02. All linear regressions used robust standard errors clustered by family identity. [a]These linear regressions excluded each parent's first offspring and were additionally adjusted for PA_j, the parent's age at the birth of their first offspring. [b]Fixed effects regressions grouped by family identity.

Regression type	Model		$\hat{\beta}_{YiPAi}$ (95% CI)
(A) No seasonality, confounding by U_j			
Linear regression	(A1)	PA_i	0.210 (0.210, 0.210)
Linear regression	(A2)	$PA_i + DOB_i$	0.143 (0.142, 0.143)
Linear regression	(A3)	$PA_i + U_j$	0.174 (0.174, 0.175)
Linear regression	(A4)	$PA_i + DOB_i + U_j$	0.100 (0.100, 0.100)
Linear regression[a]	(A5)	$PA_j + PA_i$	0.150 (0.150, 0.150)
Linear regression[a]	(A6)	$PA_j + PA_i + DOB_i$	0.110 (0.110, 0.110)
Linear regression[a]	(A7)	$PA_j + PA_i + U_j$	0.150 (0.150, 0.150)
Linear regression[a]	(A8)	$PA_j + PA_i + DOB_i + U_j$	0.100 (0.100, 0.100)
Sibling-comparison[b]	(A9)	PA_i	0.150 (0.150, 0.150)
Sibling-comparison[b]	(A10)	$PA_i + DOB_i$	0.150 (0.150, 0.150)
Sibling-comparison[b]	(A11)	$PA_i + YOB_i$	0.150 (0.147, 0.152)
Sibling-comparison[b]	(A12)	$PA_i + YOB_i$ (indicators)	0.150 (0.147, 0.152)
Sibling-comparison[b]	(A13)	$PA_i + DOB_i$(0 dp)	0.151 (0.149, 0.154)
Sibling-comparison[b]	(A14)	$PA_i + DOB_i$(1 dp)	0.153 (0.129, 0.177)
Sibling-comparison[b]	(A15)	$PA_i + DOB_i$(2 dp)	0.111 (-0.130, 0.353)
Sibling-comparison[b]	(A16)	$PA_i + DOB_i$(3 dp)	-0.627 (-3.041, 1.786)
Linear regression	(A17)	$PA_i + DOB_j$	0.191 (0.190, 0.191)
(B) Seasonality, confounding by U_j			
Sibling-comparison[b]	(B9)	PA_i	0.150 (0.150, 0.150)
Sibling-comparison[b]	(B11)	$PA_i + YOB_i$	0.111 (0.109, 0.114)
Sibling-comparison[b]	(B13)	$PA_i + DOB_i$(0 dp)	0.190 (0.187, 0.192)
Sibling-comparison[b]	(B14)	$PA_i + DOB_i$(1 dp)	0.153 (0.129, 0.177)
Sibling-comparison[b]	(B15)	$PA_i + DOB_i$(2 dp)	0.111 (-0.130, 0.352)
Sibling-comparison[b]	(B16)	$PA_i + DOB_i$(3 dp)	-0.609 (-3.022, 1.805)
(C) Seasonality, no confounding by U_j			
Linear regression	(C1)	PA_i	0.171 (0.170, 0.171)
Linear regression	(C2)	$PA_i + DOB_i$	0.100 (0.100, 0.100)
Sibling-comparison[b]	(C9)	PA_i	0.150 (0.150, 0.150)
Linear regression	(C17)	$PA_i + DOB_j$	0.150 (0.150, 0.150)
